# Natural selection and genetic diversity of domain I of *Plasmodium falciparum* apical membrane antigen-1 on Bioko Island

**DOI:** 10.1186/s12936-019-2948-y

**Published:** 2019-09-18

**Authors:** Ya-Nan Wang, Min Lin, Xue-Yan Liang, Jiang-Tao Chen, Dong-De Xie, Yu-Ling Wang, Carlos Salas Ehapo, Urbano Monsuy Eyi, Hui-Ying Huang, Jing-Li Wu, Dan-Yan Xu, Zhi-Mao Chen, Yi-Long Cao, Hai-Bin Chen

**Affiliations:** 1 Department of Histology and Embryology, Shantou University Medical College, Shantou, Guangdong People’s Republic of China; 2 School of Food Engineering and Biotechnology, Hanshan Normal University, Chaozhou, Guangdong People’s Republic of China; 3 Laboratory Medical Centre, Huizhou Municipal Central Hospital, Huizhou, Guangdong People’s Republic of China; 4 The Chinese Medical Aid Team to the Republic of Equatorial Guinea, Guangzhou, Guangdong People’s Republic of China; 5 Department of Medical Laboratory, Malabo Regional Hospital, Malabo, Equatorial Guinea; 6 2014 Clinical Medicine Programme, Shantou University Medical College, Shantou, Guangdong People’s Republic of China

**Keywords:** Bioko Island, *Plasmodium falciparum*, AMA-1, Domain I, Natural selection, Genetic diversity

## Abstract

**Background:**

*Plasmodium falciparum* apical membrane antigen-1 (*Pf*AMA-1) is a promising candidate antigen for a blood-stage malaria vaccine. However, antigenic variation and diversity of *Pf*AMA-1 are still major problems to design a universal malaria vaccine based on this antigen, especially against domain I (DI). Detail understanding of the *Pf*AMA-1 gene polymorphism can provide useful information on this potential vaccine component. Here, general characteristics of genetic structure and the effect of natural selection of DIs among Bioko *P. falciparum* isolates were analysed.

**Methods:**

214 blood samples were collected from Bioko Island patients with *P. falciparum* malaria between 2011 and 2017. A fragment spanning DI of *Pf*AMA-1 was amplified by nested polymerase chain reaction and sequenced. Polymorphic characteristics and the effect of natural selection were analysed using MEGA 5.0, DnaSP 6.0 and Popart programs. Genetic diversity in 576 global *Pf*AMA-1 DIs were also analysed. Protein function prediction of new amino acid mutation sites was performed using PolyPhen-2 program.

**Results:**

131 different haplotypes of *Pf*AMA-1 were identified in 214 Bioko Island *P. falciparum* isolates. Most amino acid changes identified on Bioko Island were found in C1L. 32 amino acid changes identified in *Pf*AMA-1 sequences from Bioko Island were found in predicted RBC-binding sites, B cell epitopes or IUR regions. Overall patterns of amino acid changes of Bioko *Pf*AMA-1 DIs were similar to those in global *Pf*AMA-1 isolates. Differential amino acid substitution frequencies were observed for samples from different geographical regions. Eight new amino acid changes of Bioko island isolates were also identified and their three-dimensional protein structural consequences were predicted. Evidence for natural selection and recombination event were observed in global isolates.

**Conclusions:**

Patterns of nucleotide diversity and amino acid polymorphisms of Bioko Island isolates were similar to those of global *Pf*AMA-1 DIs. Balancing natural selection across DIs might play a major role in generating genetic diversity in global isolates. Most amino acid changes in DIs occurred in predicted B-cell epitopes. Novel sites mapped on a three dimensional structure of *Pf*AMA-1 showed that these regions were located at the corner. These results may provide significant value in the design of a malaria vaccine based on this antigen.

## Background

Although global morbidity and mortality have decreased substantially, malaria is still a great public health concern, especially in Africa [1]. The World Malaria Report 2018 draws on data from 87 countries and areas with ongoing malaria transmission. Recent research shows that after an unprecedented period of success in global malaria control, progress has stalled. An estimated 219 million (95% confidence interval [CI]: 203–262 million) persons suffer from malaria infections worldwide, with 435,000 malaria deaths in 2017. Fifteen countries accounted for 80% of global malaria deaths in 2017, and the 10 highest burdened African countries saw an estimated 3.5 million more malaria cases in 2017 compared with the previous year [2]. Malaria is endemic in Equatorial Guinea, a country in Central West Africa with a population of around 1 million inhabitants. In 2004, the government and private parties formed a Public–Private Partnership that has spent the last 15 years controlling malaria on Bioko Island and parts of the mainland in an effort to reduce malaria’s burden on the population. The Bioko Island Malaria Control Project (BIMCP) implemented by the U.S. NGO, Medical Care Development International (MCDI) and the Ministry of Health and Social Welfare of the Government of Equatorial Guinea, has reduced malaria prevalence from 74% (by thick blood smear) in 2003 to 11% (by rapid diagnostic test) in 2017 in children 2 to 14 years of age. Infant mortality due to malaria infection has reduced by 85% (by rapid diagnostic test). However, it has proven difficult to eliminate malaria from this region despite the increasing intensity of malaria intervention. With the emergence and geographical expansion of anti-malarial resistance worldwide, molecular markers are essential tools for the surveillance of resistant *Plasmodium* parasites [3]. The decrease of island-wide *P. falciparum* prevalence was steep in the first year following the implementation of malaria intervention. However, it is still a major obstacle to public health and economic growth for countries in the tropics and subtropical regions [4].

Vaccines are the most cost effective and efficient method of protecting against malaria. However, malaria, one of the oldest and deadliest pathogens in human history, remains without a marketed vaccine. The Equato-Guinean Malaria Vaccine Initiative (EGMVI) is engaging in conducting a series of clinical trials that will advance a *Pf*SPZ vaccine through to phase III clinical trials and eventually test the public health utility of the vaccine in malaria elimination projects. Therefore, the development of an effective vaccine against *P. falciparum* is a necessary priority, particularly due to the increased resistance of this parasite to anti-malarial drugs [3, 5]. Up to date, several candidate proteins, including circumsporozoite protein (CSP), Duffy-binding protein (DBP), merozoite surface protein-1 (MSP-1), apical membrane antigen-1 (AMA-1), and thrombospondin-related anonymous protein (TRAP) have been tested for their potential as candidate antigens for the development of effective vaccines [6]. For MSP-1, previous report showed high genetic diversity and MOI values among the *P. falciparum* population [7]. A high prevalence of *Pfdhfr*-*Pfdhps* quadruple mutations were detected, which is associated with sulfadoxine resistance in *P. falciparum* isolates on Bioko Island. This result reflects both the high endemic level of malaria and its transmission on Bioko Island [8].

AMA-1 is an 83-kDa type I integral membrane protein localized to the apical complex [9]. It is mainly expressed in the merozoite and sporozoite stages of malaria parasites and appears to be transported to the merozoite surface in late schizonts and free merozoites [9–14]. AMA-1 consists of a signal sequence, a cysteine-rich ectodomain, a conserved cytoplasmic region and a transmembrane region [15]. The ectodomain of AMA-1 is subdivided into three immunogenic domains, and natural immune responses against this domain have been reported in patients infected with *P. falciparum* [16, 17]. The biological function of AMA-1 is not understood thoroughly, though its stage-specific expression and localization suggest that this protein might play a crucial role in invasion [9, 12, 18, 19]. There is also several evidence that suggest that AMA-1 forms a complex with RON2 to form the moving junction during invasion of merozoites into erythrocytes [17–23]. According to a previous report, AMA-1 can induce a protective immune response that produces antibodies that can effectively inhibit *P. falciparum* from invading red blood cells [24]. However, polymorphism of protozoan antigen and immune evasion of *plasmodium* have impeded the development of a malaria vaccine. *Pf*AMA1 is the main candidate antigen of a red-blood stage vaccine and has entered a phase II clinical trial [25]. Due to its essential role for parasite survival and high level of immunogenicity during natural infection in humans, vaccination studies conducted in BALB/C mouse models have confirmed that AMA-1 is a potential vaccine antigen against *P. falciparum* [26].

Independent studies provide strong evidence that balancing selection acts to maintain these polymorphisms in the population, reflecting the importance of AMA-1 as a target of protective immunity [27–30]. However, these antibodies can recognize either conserved or allele-specific epitopes of AMA-1, resulting in limited protection for different alleles [31]. Several polymorphisms can be observed in DI of AMA-1, suggesting that this region seems to be the main target of anti-AMA-1 protective antibodies [30, 32–35]. Nevertheless, genetic diversity of AMA-1 among *Plasmodium* field isolates and the presence of variant forms in different geographic areas present hurdles in successful malaria vaccine design. Therefore, in order to design an efficient and protective malaria vaccine, it is essential to monitor genetic variations of candidate vaccine antigens in global malaria isolates circulating in endemic areas [36].

Although the incidence of malaria has decreased significantly through BIMCP, *P. falciparum* is still the most critical concern for malaria control and prevention on Bioko Island with the emergence of artemisinin resistance [3, 5]. In this study, natural selection and genetic diversity of AMA-1 DI region in Bioko *P. falciparum* isolates were analysed.

## Methods

### Study area

**Fig. 1:**
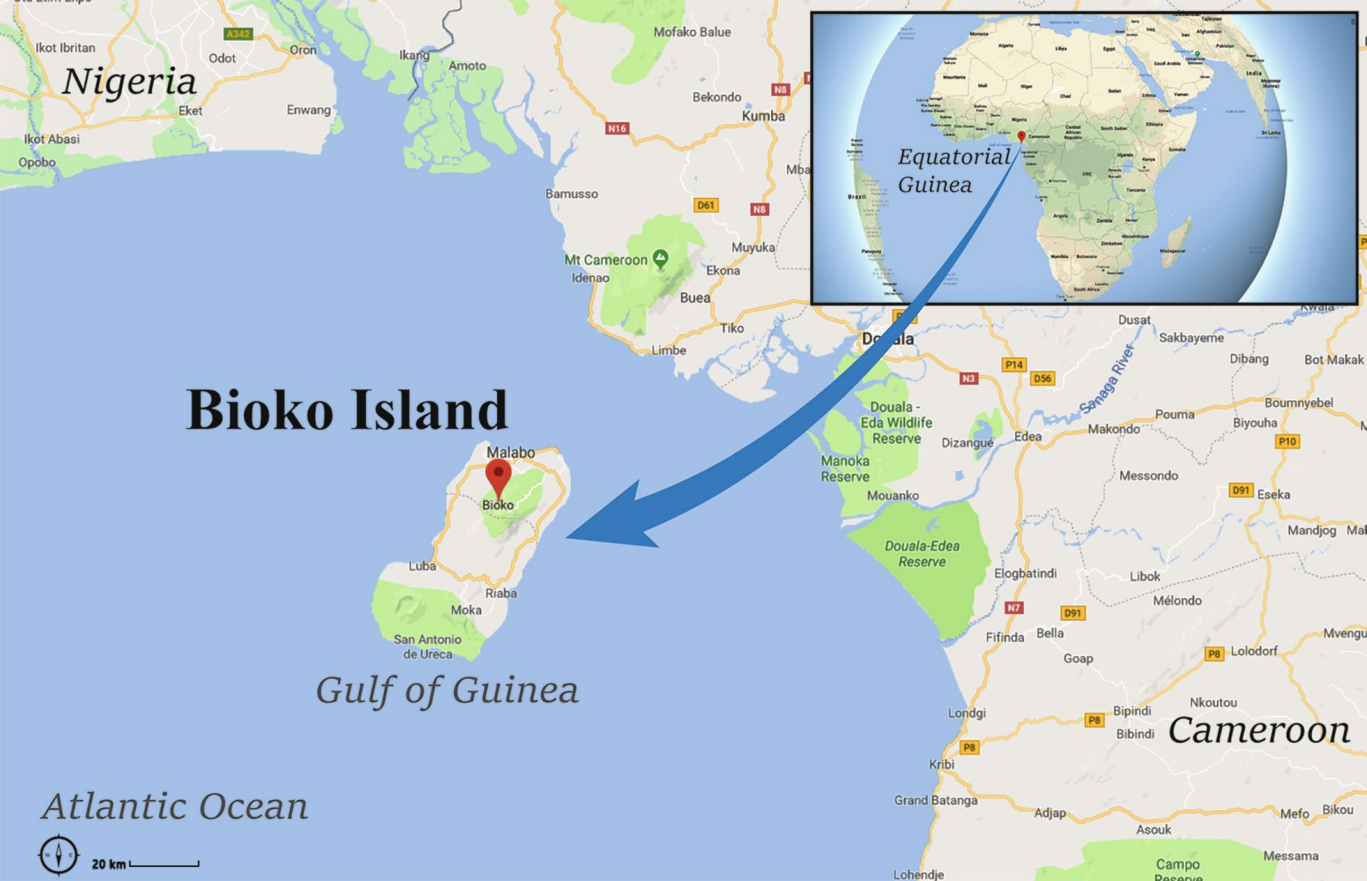
Map of Bioko Island of Equatorial Guinea

The study was carried out in the clinic of the Chinese medical aid team for the Republic of Equatorial Guinea, Malabo Regional Hospital. Ethical approval was obtained from the Ethics Committee of Malabo Regional Hospital (EGCNGD-071). Bioko Island, the largest island of Equatorial Guinea, is located in the Gulf of Guinea, approximately 100 km off the coast of southern Nigeria and 160 km northwest of continental Equatorial Guinea (Fig. 1). The island has a population of 334,463 inhabitants (2015 census, of which approximately 90% live in Malabo, the capital city of Equatorial Guinea) and a humid tropical environment.

### Sample collection

*Plasmodium falciparum* clinical samples (from individuals 4 months to 80 years of age) were collected from 214 confirmed *P. falciparum* malaria cases identified by microscopic examination and an immuno-colloidal gold test kit (ICT Diagnostics) in 2011–2017. Informed consent was obtained from all participating subjects or their parents. Blood samples were collected on filter paper (Whatman 3 mm, GE Healthcare, Pittsburg, USA) for further molecular analysis, air-dried and stored in sealed plastic bags at ambient temperature. Thick blood smears were air dried and stained with 10% fresh Giemsa following standard procedures. After coding and recording the patient medical records, the dried blood filters were then stored in plastic sealing bags and stored at − 80 °C.

### DNA extraction

Parasite DNA was extracted from dried filter blood spots by following the Chelex-100 extraction method described in previous article [3]. The DNA products were collected in sterile tubes and stored at −20 °C.

### Amplification and sequencing analysis of DI of *Pf*AMA-1

AMA-1 sequences were amplified by nested PCR. For the first round PCR, 1 μl of DNA was added to 12.5 μl 2× MasterMix (DNA polymerase, dNTP mixture, PCR buffer), 1 μl of 10 μmol/l forward primer (5′-TGAAGAAGTTCATGGTTCAGGT-3′), 1 μl of 10 μmol/l reverse primer (5′-GCACTTTTGATCATACTAGCGTT-3′), and sterile ultrapure water to a final volume of 25 μl. Thermal cycling parameters for PCR were as follows: one cycle of an initial denaturation at 94 °C for 3 min, 30 cycles of 94 °C for 30 s, annealing at 55 °C for 30 s and extension at 72 °C for 2 min, followed by a final extension step at 72 °C for 5 min. For the second round PCR, 2 μl of the primary PCR product was amplified in a 50 μl reaction volume comprised of 25 μl 2× Master Mix (DNA polymerase, dNTP mixture, PCR buffer), 2 μl of 10 μmol/l forward primer (5′-GTTGATCCGAAGCACTCA-3′), 2 μl of 10 μmol/l reverse primer (5′-AGATGCTGAAGTAGCTGGAA-3′), and sterile ultrapure water to a final volume of 50 μl. All PCR products were analysed using 2.0% agarose gel electrophoresis, and then, they were purified and sequenced by using an ABI 3730 × L Automated Sequencer (Shanghai Yingjun Biotechnology Co., LTD, Guangzhou branch). In our study, DI (nt: 445 ~ 906 bp; 149–302 aa) sequences of the *Pf*AMA-1 gene in 214 *P. falciparum* isolates were successfully amplified, including the C1 region (559–693 bp; 187–207 aa), C1L region (586–621 bp; 197–207 aa), C2 region (724–735 bp; 242–245 aa and 844–858 bp; 282–286 aa) and C3 region (514–525 bp; 172–175 aa). To ensure the accuracy of the sequencing, at least two clones of each isolate was sequenced. Sequencing primers were the reverse primers of the second round PCR, all sequences were analysed and integrated by Bioedit and MEGA5.0 software. These nucleotide sequences have been deposited at SRA under accession numbers (SRX4999365–SRX4999578).

### Statistical analysis

#### Nucleotide sequence polymorphism analysis and neutrality test

Nucleotide and predicted amino acid sequences of DI were analysed using MEGA5.0 [37]. Nucleotide sequence polymorphism analysis was conducted for 214 sequences [37]. Numbers of segregating sites (S), haplotypes (H), haplotype diversity (Hd), nucleotide diversity (π), and average number of pair-wise nucleotide differences within a population (K) were estimated using DnaSP6.0 [38]. The value of π was calculated to estimate step-wise diversity throughout DI based on a sliding window of 100 bases with a step size of 5 bp. Values of non-synonymous (dN) and synonymous (dS) substitutions were estimated and compared using the Z test (*P* < 0.05 was considered significant) in the MEGA5.0 program [37] based on method of Nei and Gojobori [39] with Jukes and Cantor correction. Tajima’s D value [40] and Fu and Li’s D and F values [41] were analysed using DnaSP 6.0 to evaluate the neutral theory of evolution [38]. Recombination parameters (R), which included the effective population size and probability of recombination between adjacent nucleotides per generation, and minimum number of recombination events (Rm) were analysed by DnaSP6.0 [38]. Linkage disequilibrium (LD) between different polymorphic sites was computed based on the R^2^ index using DnaSP6.0 [38]. Protein function prediction of new amino acid mutation sites was performed using the PolyPhen-2 program [42].

#### Population diversity of *Pf*AMA-1 DIs among other global *P. falciparum* isolates

Genetic diversity of *Pf*AMA-1 DIs in other global *P. falciparum* isolates was analysed. Parasite populations from Ghana (n = 37, AB71569–AB715734), Tanzania (n = 62, AB715636–AB715697), Nigeria (n = 51, AJ408300–AJ408350), Solomon (n = 50, AB715960–AB716009), Gambia (n = 114, FJ555752–FJ555865), Kenya (n = 129, FN869569–FN869697), Benin (n = 23, AJ271168–AJ271190), Thailand (n = 80, AB715735–AB715814), and Venezuela (n = 30, EU332414–EU332443) were included in the analysis. All publicly available sequences covered the DI sequences of *Pf*AMA-1. Nucleotide sequence polymorphism analysis and neutrality test for each population were performed using programs DnaSP6.0 [38] and MEGA5.0 [37] as described above. Genetic differentiation among parasite populations was calculated based on the fixation index (Fst) to estimate pairwise DNA sequence diversity between and within populations using Arlequin 3.5 [43]. To investigate relationships among *Pf*AMA-1 haplotypes, the haplotype network for a total of 790 *Pf*AMA-1 sequences, including 214 Bioko Island sequences and the 576 publicly available sequences from Benin, Gambia, Ghana, Kenya, Nigeria, Solomon, Tanzania, Thailand and Venezuela, was constructed using the Median Joining algorithm of Popart program [44]. To assess whether genetic diversity in DI within *P. falciparum* isolates was associated with host immune pressure, genetic diversity in predicted B-cell epitopes, intrinsically unstructured/disordered regions (IUR), and RBC binding regions in other global *Pf*AMA-1 were analysed [45–48]. Nucleotide diversity and natural selection of each region were analysed using DnasP6.0 as described above [38].

## Results

### Sequence polymorphism in DI sequences of *Pf*AMA-1 on Bioko Island

**Fig. 2:**
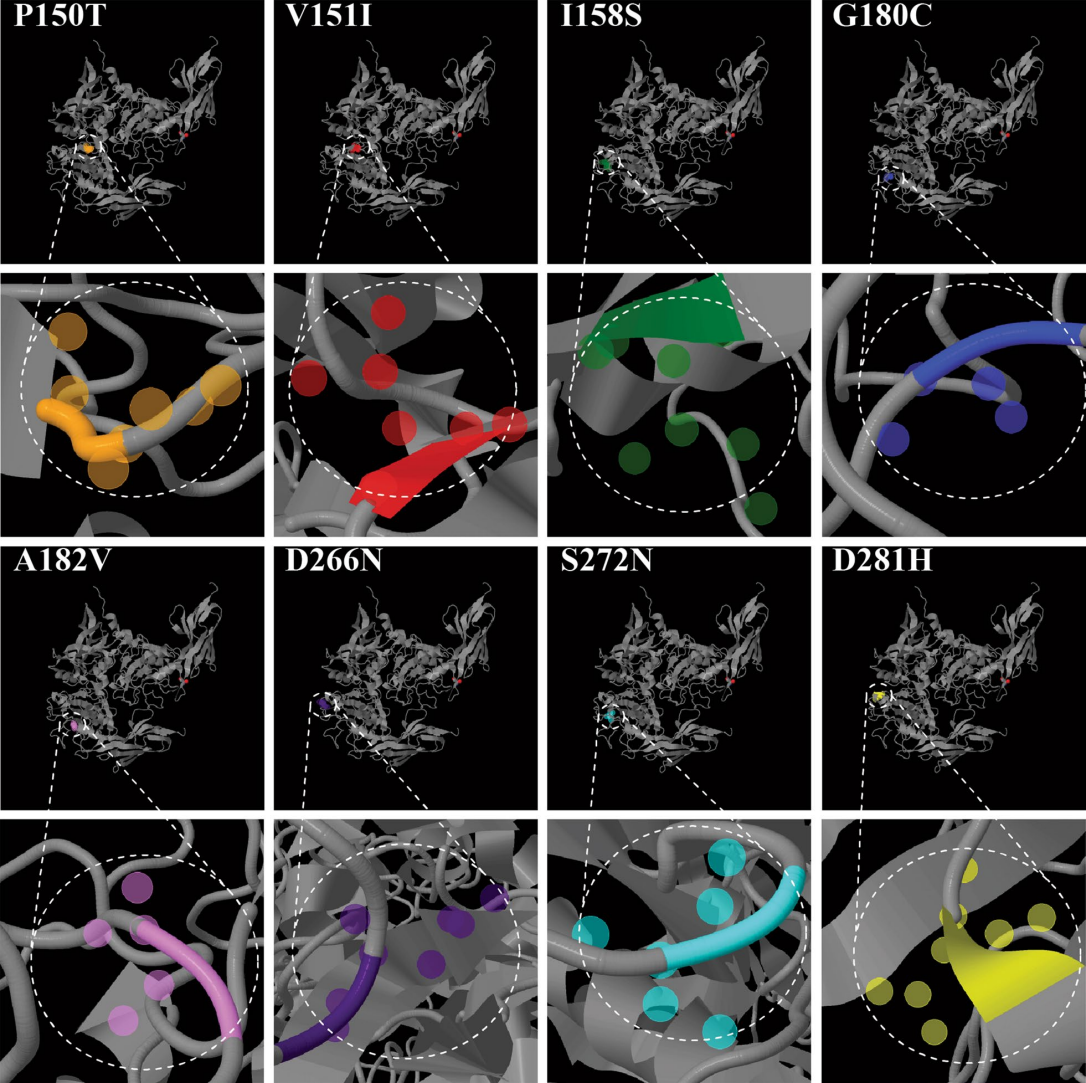
Predicted three-dimensional structure of 8 novel sites

One hundred thirty-one different haplotypes of DI were identified in 214 Bioko *P. falciparum* isolates. Comparison of 214 DI sequences with *Pf*AMA-1 sequences from the 3D7 *P. falciparum* clone (GenBank Accession Number U65407) revealed amino acid changes at 40 positions in 214 Bioko *Pf*AMA-1 sequences, including 26 di-morphic amino acid changes and 14 tri-, tetra-, or penta-morphic amino acid changes (Additional file 1). Most amino acid changes were in C1 region (14 amino acid changes), and these amino acid changes were distributed throughout each Bioko *Pf*AMA-1 haplotype. Of the 14 tri-, tetra-, or penta-morphic amino acid changes, eight (E187K/N/D, L189P/H, D196N/K, E197Q/G/H/D/R/C, H200L/D/R/V/G, F201L/S, D204 N/K and K230Q/E) were found in C1 region, whereas others (C149K/Y/*, G172E/V, Y175D/H, K243E/N, K245E/N, I282K/E) were found primarily in the C2 and C3 regions. Among 26 dimorphic amino acid changes identified in Bioko *Pf*AMA-1 isolates, 18 have been previously reported in other geographical *P. falciparum* isolates. However, the remaining 8 changes P150T (2.29%), V151I (0.76%), I158S (1.53%), G180C (2.29%), A182V (0.76%), D266N (3.82%), S272N (0.76%) and D281H (1.53%) were novel (Additional file 1). Function prediction, using the PolyPhen-2 program, of the 8 novel sites showed that all 8 mutations were likely to affect the structure and function of *Pf*AMA-1 (Table 5). The protein structural bioinformatics analysis inferred that these 8 mutations distributing the corner area of *Pf*AMA-1 protein (Fig. 2).

### Amino acid polymorphisms in DIs of Bioko Island *Pf*AMA-1 compared to global *Pf*AMA-1 isolates

**Fig. 3:**
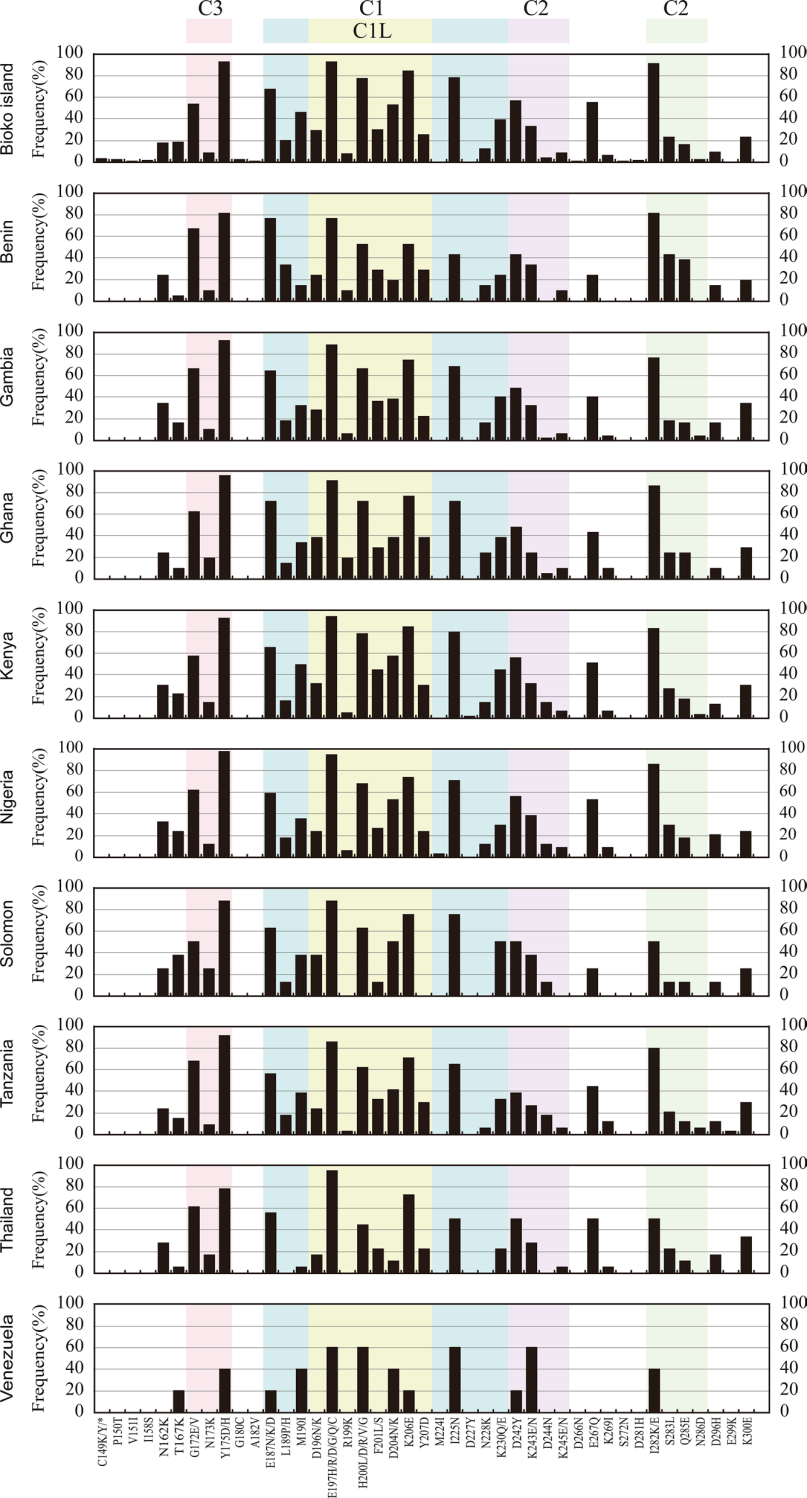
Amino acid polymorphisms of *Pf*AMA-1 among global *Plasmodium falciparum* isolates. Each region of domain I is marked by a different color: C1 region (blue), C1L region (yellow), C2 (purple), C2 (green) and C3 (pink)

When patterns of amino acid polymorphisms identified in DI of Bioko Island *Pf*AMA-1 isolates were compared to those from other countries, similar but not identical polymorphic patterns were observed. Most amino acid changes of DI, previously identified in the global *Pf*AMA-1 sequence databank, were found in the C1 region (Fig. 3). Most notable amino acid changes identified in Bioko *Pf*AMA-1 DI were Y175D/H (92.37%), E197H/R/D/G/Q/C (92.37%), which present in Bioko Island sequences commonly, meanwhile their frequencies were also high in other global *Pf*AMA-1 isolates. Frequencies of Y175D/H variation in other global isolates were as follows: Benin (80.95%), Gambia (92.00%), Ghana (95.24%), Kenya (92.06%), Nigeria (97.06%), Solomon (87.50%), Tanzania (91.18%), Thailand (77.78%), and Venezuela (40.00%). Substitutions E197H/R/D/G/Q/C were also high in C1L region from most countries: Benin (76.19%), Gambia (88.00%), Ghana (90.48%), Kenya (93.65%), Nigeria (94.12%), Solomon (87.50%), Tanzania (85.30%), and Thailand (94.45%), but not Venezuela (60.00%). High frequencies of H200D/L/R/V/G (77.09%), K206E (83.97%), I225 N (77.86%) and I282 K/E (90.83%) variations were also observed in C1 and C2 regions of Bioko *Pf*AMA-1 isolates. Highly polymorphic patterns of these four amino acid changes were also present in other global *Pf*AMA-1 isolates. Two amino acid changes (D244 N, N286D) in C2 region were common in some of the African *Pf*AMA-1 sequences (Gambia, Kenya, and Tanzania), but they were not identified or very rarely identified in the DI region from other countries. Moreover, P150T, V151I, I158S, G180C, A182 V, D266 N, S272 N, D281H mutations were uniquely identified in Bioko *Pf*AMA-1 isolates, although their substitution frequencies were very low.

### Nucleotide diversity and natural selection of DIs from Bioko *P. falciparum* isolates

Nucleotide diversity and natural selection of DIs from 214 Bioko *P. falciparum* isolates were analysed (Table 1). The K value was 12.795. The highest nucleotide differences were found in C1 region (K = 7.489), whereas the lowest were found in C3 region (K = 0.925). Haplotype diversity for DIs was 0.991 ± 0.0017. This value was higher for the C1 region (0.976 ± 0.003) than for the C2 (0.599 ± 0.031 and 0.777 ± 0.018) or C3 (0.692 ± 0.020) region. The π value of DIs was 0.02776 ± 0.00042. Analysis of π values for the C1, C1L, C2, and C3 regions revealed that the most nucleotide diversity was concentrated in the C1L and C2 regions. To examine whether natural selection has contributed to generation of DI diversity in Bioko *P. falciparum* populations, the value of dN − dS was estimated using the Nei and Gojobori method. The value of dN − dS for DIs was 0.0327, suggesting that balancing natural selection might have occurred in DIs of the Bioko *P. falciparum* populations. Considering high positive dN − dS values for the C1L (0.1378) and C2 (0.1669) regions, these regions might experience the most pressure from balancing natural selection forces. The estimated Tajima’s D value of DIs was 0.56734 (*P* > 0.10). When Tajima’s D value was analysed for each domain, the C1 (1.49861, *P* > 0.10) and C1L (1.24277, *P* > 0.10) regions showed higher positive Tajima’s D values compared to other regions.

**Table 1:** DNA sequence polymorphism and tests of neutrality at *Pf*AMA-1 among *Plasmodium falciparum* Bioko Island isolates

Fragment	Nt/bp	S	Total no. of mutations	K	H	Hd ± SD	π ± SD	dN − dS	Tajima’s D
Domain I	445–906	56	64	12.795	131	0.9911 ± 0.0017	0.02776 ± 0.00042	0.0327	0.56734 (P > 0.10)
C1	559–693	23	29	7.489	87	0.976 ± 0.003	0.05548 ± 0.00084	0.0641	1.49861 (P > 0.10)
C1L	586–621	15	18	4.469	52	0.937 ± 0.006	0.12413 ± 0.00214	0.1378	1.24277 (P > 0.10)
C2	724–735	6	6	1.421	11	0.777 ± 0.018	0.11840 ± 0.00490	0.1669	0.82057 (P > 0.10)
	844–858	5	5	0.957	6	0.599 ± 0.031	0.06381 ± 0.00416	0.0834	0.26245 (P > 0.10)
C3	514–525	3	5	0.925	7	0.692 ± 0.020	0.07711 ± 0.00361	0.1018	0.19039 (P > 0.10)

*S* segregating sites,* K* average number of pairwise nucleotide differences,* H* number of haplotypes,* Hd* haplotype diversity,* π* observed average pairwise nucleotide diversity,* dN* rate of non-synonymous mutations,* dS* rate of synonymous mutations

* P < 0.05, ** P < 0.02

### Nucleotide diversity and natural selection of DIs in other global *P. falciparum* isolates

**Fig. 4:**
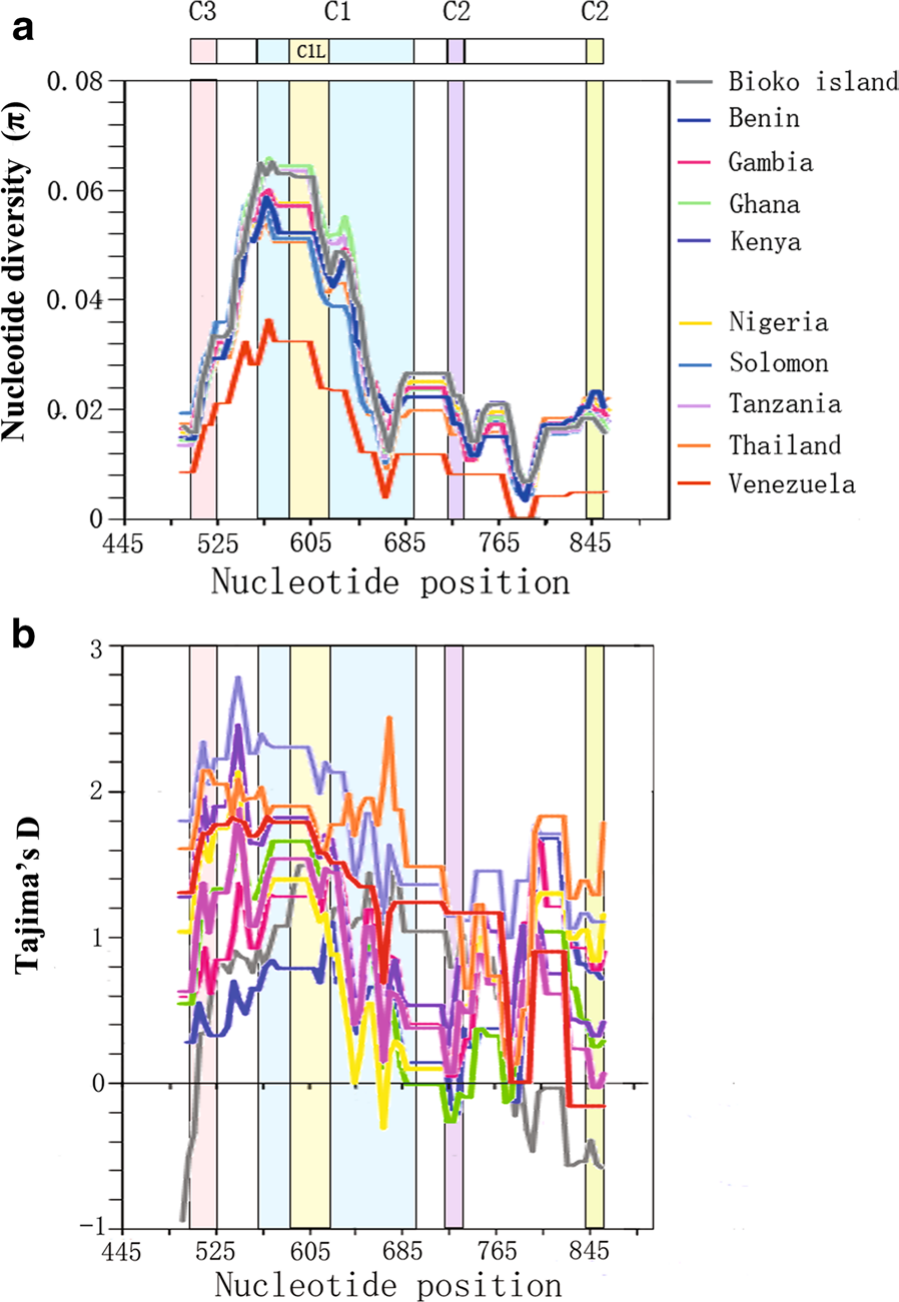
Nucleotide diversity and natural selection of DIs of global *Pf*AMA-1 sequences. **a** Nucleotide diversity. Sliding window plot analysis shows the nucleotide diversity (π) value across DIs of other global *Pf*AMA-1 sequences. A window size of 100 bp and a step size of 5 bp were used. **b** Natural selection. Sliding window calculation of Tajima’s D statistic was performed for global *Pf*AMA-1 genes. A window size of 100 and a step size of 5 were used. Bioko Island, jasper; Benin, light blue; Gambia, pink; Ghana, green; Kenya, deep blue; Nigeria, yellow; Solomon, purple; Tanzania, gray; Thailand, orange; Venezuela, red

Nucleotide diversity of DIs among other global isolates, including Benin, Gambia, Ghana, Kenya, Nigeria, Solomon, Tanzania, Thailand and Venezuela were analysed, and compared to Bioko *Pf*AMA-1 isolates. K values of DIs among African *Pf*AMA-1 isolates (Ghana, K = 12.555; Kenya, K = 12.886) were higher than those of Asian (Thailand, K = 11.428) and South American (Venezuela, K = 5.617) *Pf*AMA-1 isolates (Table 2). Nucleotide diversity across DIs from different countries also slightly differed by geographical area. The level of nucleotide diversity across the DIs of Bioko Island *P. falciparum* isolates (π = 0.02776) was higher than that from Venezuela (π = 0.01216) or Thailand (π = 0.02474) isolates, but similar to that from Ghana (π = 0.02717), Benin (π = 0.02577), Tanzania (π = 0.02697) and Solomon (π = 0.02552). A sliding window plot of π revealed that DIs from different geographical areas shared highly similar patterns of nucleotide diversity through their sequences, with two peaks in the C1 region. Maximum diversity was found in the C1 region (Fig. 4a). All DI sequences from different countries showed positive Tajima’s D values, suggesting a pattern of balancing selection across DIs in global *P. falciparum* samples (Table 2). A sliding window plot analysis also showed that DIs of other global *Pf*AMA-1 genes had a similar pattern for Tajima’s D across the board, albeit some differences were identified among DIs with different geographical origins (Fig. 4b).

**Table 2:** Estimates of DNA sequence polymorphism and tests of neutrality at P*f*AMA-1 among other global *plasmodium falciparum* isolates

Isolate	S	Total no. of mutations	K	H	Hd ± SD	π ± SD	Tajima’s D	Fu and Li’s D	Fu and Li’s F
Bioko Island (n = 214)	56	64	12.795	131	0.9911 ± 0.0017	0.02776 ± 0.00042	0.56734 (P > 0.10)	− 0.27228 (P > 0.10)	0.12925 (P > 0.10)
Ghana (n = 37)	37	41	12.555	22	0.967 ± 0.012	0.02717 ± 0.00090	1.01706 (P > 0.10)	1.29910 (P > 0.10)	1.42603 (P > 0.10)
Tanzania (n = 62)	40	45	12.460	35	0.972 ± 0.008	0.02697 ± 0.00061	1.01442 (P > 0.10)	1.59904 (P < 0.05)	1.64786 (0.10 > P > 0.05)
Nigeria (n = 51)	38	42	12.409	35	0.979 ± 0.008	0.02686 ± 0.00068	1.13803 (P > 0.10)	1.53256 (P < 0.05)	1.65608 (0.10 > P > 0.05)
Solomon (n = 50)	31	32	11.791	9	0.844 ± 0.021	0.02552 ± 0.00074	2.19486 (P < 0.05)	1.34414 (0.10 > P>0.05)	1.96178 (P < 0.02)
Gambia (n = 114)	42	48	12.267	51	0.970 ± 0.006	0.02655 ± 0.00051	1.12103 (P > 0.10)	1.22880 (P > 0.10)	1.42163 (P > 0.10)
Kenya (n = 129)	42	48	12.886	64	0.980 ± 0.004	0.02789 ± 0.00038	1.42081 (P > 0.10)	1.71986 (P < 0.02)	1.91516 (P < 0.05)
Benin (n = 23)	34	37	11.605	22	0.993 ± 0.014	0.02577 ± 0.00237	0.65437 (P > 0.10)	1.22364 (P > 0.10)	1.22668 (P > 0.10)
Venezuela (n = 30)	15	15	5.617	6	0.482 ± 0.101	0.01216 ± 0.00228	1.65975 (P > 0.10)	1.14686 (P > 0.10)	1.53677 (0.10 > P > 0.05)
Thailand (n = 80)	32	34	11.428	19	0.919 ± 0.012	0.02474 ± 0.00067	2.11203 (P < 0.05)	0.83255 (P > 0.10)	1.58398 (0.10 > P > 0.05)

*S* segregating sites,* K* average number of pairwise nucleotide differences,* H* number of haplotypes,* Hd* haplotype diversity,* π* observed average pairwise nucleotide diversity

### Recombination and linkage disequilibrium

The minimum number of recombination events between adjacent polymorphic sites (Rm) for DIs of Bioko *Pf*AMA-1 was estimated to be 21. The R values between adjacent sites (Ra) and per gene (Rb) were 0.2646 and 122, respectively. Possible recombination events were also identified in DIs of other global *Pf*AMA-1 genes. The highest R values were predicted for DIs of African *Pf*AMA-1 (Nigeria and Kenya) populations, whereas the lowest R values were predicted for DIs of Venezuela *Pf*AMA-1 isolates (Table 3). The LD index (R^2^) for DIs of global *Pf*AMA-1 genes also decreased with increasing distance across this gene (Additional file 2).

**Table 3:** Comparison of recombination events of domain I between other global P*f* AMA-1genes

	Ra	Rb	Rm
Bioko Island	0.2646	122	21
Ghana	0.1813	83.6	12
Tanzania	0.2191	101	12
Nigeria	0.2321	107	13
Solomon	0.0397	18.3	6
Gambia	0.1666	76.8	15
Kenya	0.2321	107	16
Benin	0.0865	39.9	10
Venezuela	0.000	0.001	1
Thailand	0.0751	34.6	8

The R and Rm were estimated excluding the sites containing alignment gaps or those segregating for three nucleotides. The R was computed using R = 4Nr, where N is the population size and r is the recombination rate per sequence (per gene) n, number of isolates; Ra, recombination parameter between adjacent sites; Rb, recombination parameter for entire gene; Rm, minimum number of recombination events between adjacent sites

### Nucleotide differentiation among DIs of other global *Pf*AMA-1

To investigate the degree of gene flow and genetic differentiation among DIs of global *Pf*AMA-1 sequences, Fst values were evaluated for DIs from different geographical *P. falciparum* populations deposited in GenBank (Table 4). Fst values between different geographical *Pf*AMA-1 populations varied from 0.00317 (−, P > 0.05) between Kenya and Nigeria to 0.32747 (+, P < 0.05) between Venezuela and Benin, except negative values. Three negative values appeared in the Fst analysis, which may be due to the close geographical location of the sample source and the short sequence interval analysed in this study.

**Table 4:** Pairwise Fst estimates for DI of P*f*AMA-1

	Bioko Island	Benin	Gambia	Tanzania	Ghana	Nigeria	Kenya	Thailand	Venezuela	Solomon
Bioko Island		**+**	**+**	**+**	**–**	**–**	**+**	**+**	**+**	**+**
Benin	0.04597		**–**	**+**	**–**	**+**	**+**	**+**	**+**	**+**
Gambia	0.01824	0.01760		**–**	**–**	**–**	**–**	**+**	**+**	**+**
Tanzania	0.01218	0.03052	0.00330		**–**	**–**	**–**	**+**	**+**	**+**
Ghana	0.00548	0.01743	0.00765	− 0.00464		**–**	**–**	**–**	**+**	**+**
Nigeria	0.00812	0.05139	0.00582	0.00439	0.01375		**–**	**+**	**+**	**+**
Kenya	0.00850	0.03536	0.00809	− 0.00623	− 0.00267	0.00317		**+**	**+**	**+**
Thailand	0.03965	0.03866	0.03152	0.03677	0.01610	0.03908	0.03506		**+**	**+**
Venezuela	0.18281	0.32747	0.20991	0.23285	0.25141	0.20272	0.20219	0.23502		**+**
Solomon	0.05101	0.07254	0.03205	0.04926	0.05055	0.04079	0.04068	0.04134	0.18970	

Fst values are shown in the lower left quadrant and P values ( +: P < 0.05 ) are shown in the upper right quadrant. Fst, a measure of genetic differentiation between populations (range from 0 to + 1).

### Haplotype network analysis

**Fig. 5:**
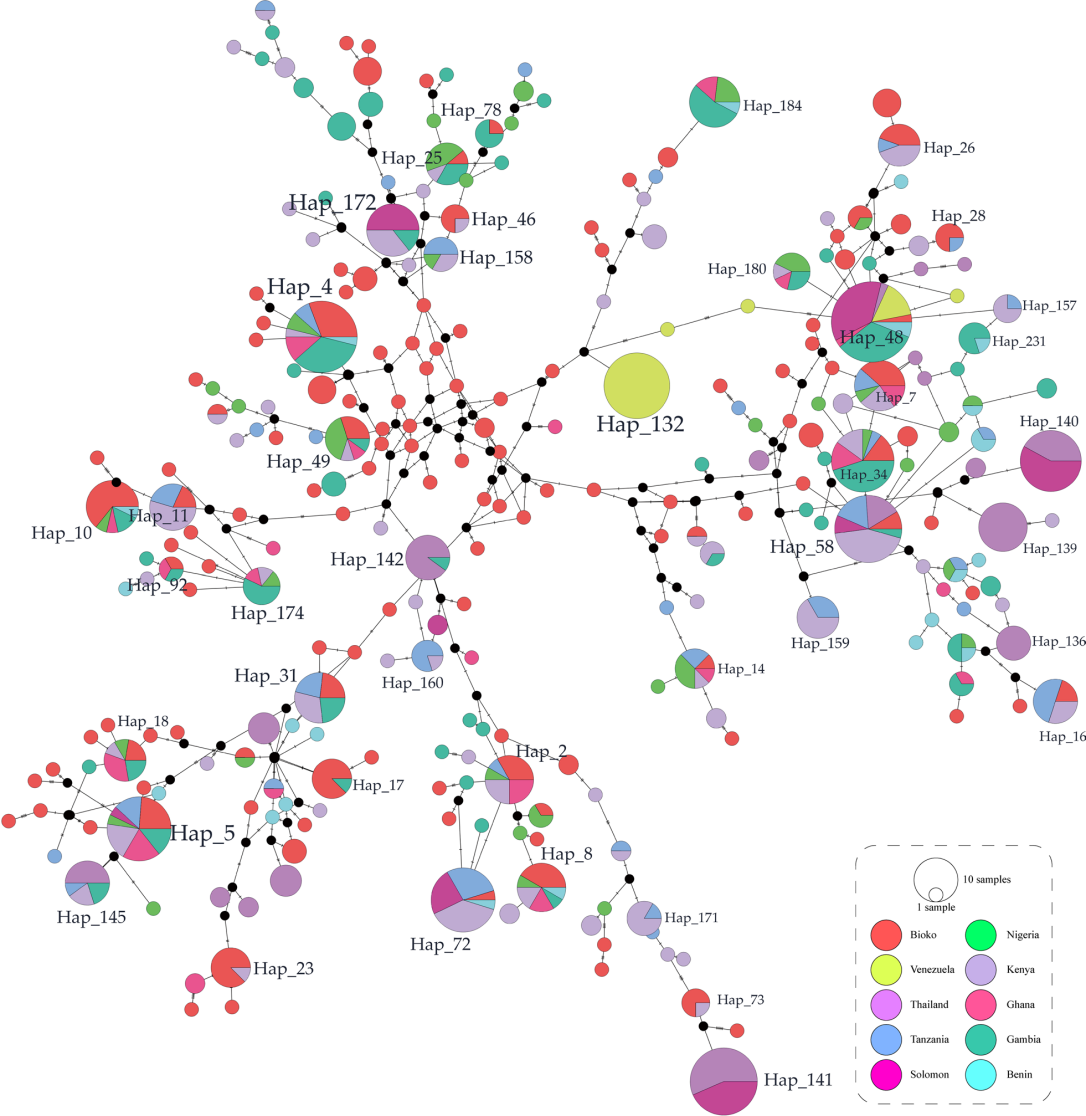
Network analysis of global *Pf*AMA-1 haplotypes. Bioko Island, yellow; Benin, light blue; Gambia, orange; Ghana, green; Kenya, light pink; Nigeria, deep blue; Solomon, red; Tanzania, deep pink; Thailand, brown; Venezuela, jasper

Haplotype network analysis of *Pf*AMA-1 haplotypes from global *P. falciparum* populations showed a dense network with pretty complex relationships (Fig. 5). A total of 268 haplotypes were identified in 790 *Pf*AMA-1 sequences, of which 66.04% was singleton. Haplotype prevalence ranged from 0.12 to 4.17%. The most prevalent haplotype was haplotype 48 (Hap_48) with a frequency of 4.17%. Haplotypes 4, 5, 34, 58, 72, 132 and 141 (Hap_4, 5, 34, 58, 72, 132 and 141) were other major haplotypes with high prevalence (2.53 to 3.29%). Only Haplotype_48 (Hap_48) contained haplotypes from three continents. Haplotypes_58, 140, 141, 142 and 145 (Hap_58, 140, 141, 142 and 145) were composed of haplotypes from two continents (Asian and African populations). Haplotypes from Venezuela were more likely to be distributed alone (Hap_132, 133, 134 and 135). Haplotypes from Bioko Island were mostly scattered with no particular distribution pattern (red pie, Fig. 5).

### Association between natural selection and host immune pressure

**Fig. 6:**
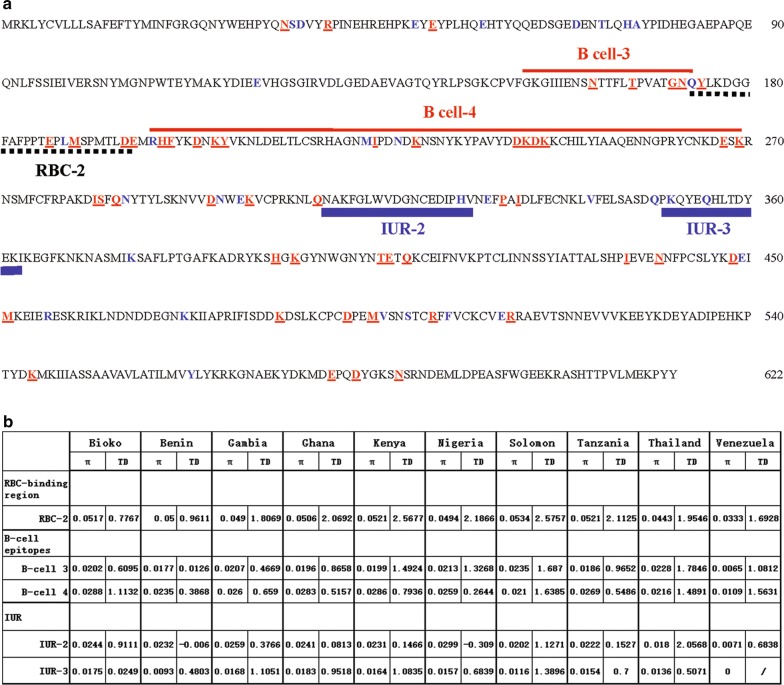
Association between natural selection and host immune pressure. **a** Positions of amino acid changes found in global PfAMA-1 and predicted RBC-binding sites, B-cell epitopes and IUR regions. Predicted RBC-binding sites, B-cell epitopes and IUR regions are presented by dotted black lines, red lines and bold blue lines, respectively. Polymorphic amino acid residues commonly identified in global PfAMA-1 are marked as bold red with underline. The less commonly identified amino acid changes are shown as bold blue. **b** Nucleotide diversity and natural selection analysis. Nucleotide diversity (π) and Tajima’s D (TD) values for each RBC-binding sites, B-cell epitopes and IUR regions in DI were analysed using Dnasp 6.0 program

The selective pressure of host immunity on DIs was evaluated by analyzing genetic polymorphisms in predicted RBC-binding sites, B-cell epitopes and IUR regions. Results showed that most amino acid changes caused by SNPs were found in predicted RBC-binding sites, B-cell epitopes or IUR regions of *Pf*AMA-1 (Fig. 6a). Seven amino acid changes were found in predicted RBC-2 binding sites, two of which (Y175D/H (92.37%), E187 N/K/D (67.18%)) were high frequency and two (G180C, A182 V) were novel sites. Five and sixteen amino acid changes were found in predicted B cell-3 and 4 regions, respectively. Most of them were commonly identified in global *Pf*AMA-1 isolates, each of these two predicted regions has a new amino acid change, I158S was in the predicted B cell-3 region and D266 N was in the predicted B cell-4 region. Also, four amino acid changes were found in the predicted IUR-2 (I225 N, N228 K, K230Q/E and D242Y) and IUR-3 (D266 N, E267Q, K269I and S272 N) regions, respectively, and were all commonly identified in global *Pf*AMA-1. The π value and Tajima’s D value of these regions were calculated in the different countries (Fig. 6b). In particular, high levels of π were predicted for RBC-2 region, and the value varied little by different country except Venezuela (0.0333). Tajima’s D values for the predicted RBC-2, B cell-3, 4 and IUR-3 regions were all positive, indicating balancing selection. Meanwhile, Tajima’s D values for predicted IUR-2 were negative (Benin and Nigeria) (Fig. 6b). The several predicted regions contained major polymorphic amino acid changes in global *Pf*AMA-1 isolates, and five of eight novel sites were found in these regions (Fig. 6a).

## Discussion

The World Malaria Report 2018 draws on data from 87 countries and areas with ongoing malaria transmission. These reports show that after an unprecedented period of success in global malaria control, progress has stalled [2]. This study provides useful information for the prevention and control of *P. falciparum* on Bioko Island by analyzing the DI region of AMA-1 gene for a *P. falciparum* vaccine, as well as characterize the genetic polymorphism and molecular evolution. Although the frequencies of these new mutation sites are not very high, four of them are distributed in B cell-3 (I158T), RBC-2 (G180C, A182V) and B cell-4 (D266N), respectively. The predicted results showed that all 8 mutations are likely to affect the structure and function of *Pf*AMA-1 (probably damaging). Most of these proteins are distributed in the corner area. Whether these mutations cause changes in protein structure and function and affect the binding with human host protein remains to be verified (Table 5, Fig. 2). Moreover, it was found that mutations in DI domain, especially in the C1, C1L, C2 and C3 regions, are highly correlated with the pathogenicity of the host after parasite antigen escape and infection [49]. Nucleotide sequence analysis of these 214 *Pf*AMA-1 sequences from Bioko populations compared to *Pf*AMA-1 from *P. falciparum* clone 3D7 (GenBank Accession Number U65407) revealed 131 different haplotypes. Most amino acid changes identified from Bioko *Pf*AMA-1 isolates were clustered in C1 region, especially the C1L region, which is consistent with previous reports on the DI region mutations of *Pf*AMA-1 from other endemic areas [45, 50]. Overall distribution patterns and frequencies of amino acid changes found on Bioko Island were similar to those of other *Pf*AMA-1 changes found globally, but several differences between Bioko Island and other global *Pf*AMA-1 isolates have also been identified in this study. The implication of these geographic differences is not entirely clear. Considering that only the DI region and a limited number of *Pf*AMA-1 isolates in each geographical area were analysed in this study, these amino acid changes and their different frequencies may not be statistically significant. In fact, many of these amino acid changes in other *Pf*AMA-1 genes were generally found and distributed globally in other *Pf*AMA-1 sequences, although their frequencies varied between and among populations. Therefore, to better understand the polymorphism of nucleotide and amino acid of *Pf*AMA-1 on Bioko Island, more *Pf*AMA-1 sequences are required.

**Table 5:** Function prediction scores of eight novel sites

Novel sites	Frequency (%)	Score (HumDiv)^a^	Sensitivity	Specificity	Classification^b^
P150T	2.29	1.000	0.00	1.00	Probably damaging
V151I	0.76	0.714	0.86	0.92	Probably damaging
I158S	1.53	0.998	0.27	0.99	Probably damaging
G180C	2.29	1.000	0.00	1.00	Probably damaging
A182V	0.76	1.000	0.00	1.00	Probably damaging
D266N	3.82	0.991	0.71	0.97	Probably damaging
S272N	0.76	0.846	0.83	0.93	Probably damaging
D281H	1.53	1.000	0.00	1.00	Probably damaging

^**a**^HumDiv is preferred model for evaluating rare alleles, dense mapping of regions identified by genome-wide association studies, and analysis of natural selection

^**b**^Qualitative ternary classfication appraised at 5%/10% (HumDiv) FPR thresholds (“benign”, “possibly damaging”, “probably damaging”)

The π value for global *Pf*AMA-1 varies, ranging from 0.01216 (Venezuela) to 0.02789 (Kenya). The π values of African *Pf*AMA-1 sequences (Bioko Island, Ghana, Tanzania, Nigeria, Gambia, Kenya and Benin) are higher than those in South American and Asian *Pf*AMA-1 sequences. The π values are higher on Bioko Island (0.02776) than in other African countries except Kenya (0.02789). Indeed, nucleotide diversity was not distributed throughout Bioko Island *Pf*AMA-1. The C2 (844–858 bp) and C3 (514–525 bp) regions show low levels of nucleotide diversity, indicating that these regions might be more conserved, with a lower frequency of polymorphisms. Moreover, much higher values of nucleotide diversity are observed in the C1L (586–621 bp) and another C2 region (724–735 bp) regions, indicating that a higher frequency of polymorphisms occurs at these regions. Indeed, DI, especially the C1 and C1L regions, are targets of the host’s immune system. The high number of DI polymorphisms in the Bioko Island population suggests that this region is under the selection of host immune pressure during evolution. Previous studies on disease-blocking vaccines using *Pf*AMA-1 monoclonal antibody showed that amino acid mutations in the C1 region (197/200/201/204/225) of *Pf*AMA-1 could block the binding of monoclonal antibody to *Pf*AMA-1, thereby inhibiting the effectiveness of the vaccine. In this study, the C1 region showed a high number of polymorphisms at the gene and amino acid levels, suggesting that the above region may affect the effectiveness of vaccines. Although the values are different among and between other global *Pf*AMA-1 isolates, highly similar distribution patterns of nucleotide diversity have been detected in global *Pf*AMA-1 genes analysed in this study, strongly indicating that these *Pf*AMA-1 genes might share highly similar nucleotide diversity. The dN − dS value for Bioko Island *Pf*AMA-1 DIs is positive, suggesting involvement of balancing selection. Also, the dN − dS value for all regions of DI is positive, indicating positive natural selection throughout DIs. The positive Tajima’s D values for Bioko Island *Pf*AMA-1 also suggested that this gene might have evolved under balancing selection. In the sliding plot analysis of Tajima’s D, general patterns of Tajima’s D values across Bioko Island *Pf*AMA-1 were similar to those of other *Pf*AMA-1 genes, although differences among and between *Pf*AMA-1s from different countries were also found. Regardless of the slight difference in values, C1, C2 and C3 regions all shared pretty similar distribution patterns of Tajima’s D values, strongly indicating that these regions might be major targets for the host immune response. Fu and Li’s D and F tests also provide enough evidence for balancing selection of *Pf*AMA-1 on Bioko Island. These values suggest that *Pf*AMA-1 DIs from Bioko Island populations are highly polymorphic with a strong selection force acting on it, similar to other global *Pf*AMA-1 genes. In addition to natural selection, recombination also contributes to the diversity of *Pf*AMA-1. Meiotic recombination that occurs between the adjacent polymorphic sites is responsible for the high allelic diversity in DI [45]. These results also indicate that a high level of recombination events have occurred in *Pf*AMA-1 isolates on Bioko Island. High levels of recombination in *Pf*AMA-1 from different geographical isolates have been previously reported [28, 35, 45, 46, 50–54]. High recombination events were found in Bioko Island *Pf*AMA-1 sequences compare to samples from other geographical areas. This might be due to special island location of Bioko region, which attribute an opportunity to *Pf*AMA-1 to undergo more recombination. Moreover, the incidence of recombination events in African countries is also generally higher than other areas, confirming the previous conclusions of recombination events. This is supported by the decline of the LD index R^2^ with increasing nucleotide distance in *Pf*AMA-1 isolates on Bioko Island, consistent with previous reports [28, 45, 46, 50–54]. In conclusion, recombination is the most important factor generating genetic diversity in global *Pf*AMA-1 populations.

The Fst value is one of the most useful methods for analyzing overall genetic differentiation (range from 0 to + 1). Fst values at each locus are considered as having no differentiation (0), low genetic differentiation (0–0.05), moderate differentiation (0.05–0.15) or high differentiation (0.15–0.25) [54]. The Fst values for *Pf*AMA-1 DIs on Bioko Island show a lower level of genetic differentiation than that of *Pf*AMA-1 in other geographical populations except Benin, which occurs at a moderate level, as well as the values of Fst for most Asian and African countries. Also, a high level of genetic differentiation is found between Bioko Island and Venezuela. All of these indicate that Fst values between *Pf*AMA-1 populations belonging to the same geographical area are relatively low. The negative values in the table were considered because DI of *Pf*AMA-1 is too limited. However, Bioko Island shows a high Fst value for Venezuela (0.22087), providing strong evidence for geographical isolation and population segmentation in these regions. Total Fst values of DI between and among global *Pf*AMA-1 isolates are in the low to medium differentiation range, indicating that *Pf*AMA-1 has limited genetic differentiation among other parasite populations around the world.

It is important to understand the haplotype network of *Pf*AMA-1 on Bioko Island with other global areas in order to develop a globally effective malaria vaccine based on this gene. A total of 296 different haplotypes were identified by network analysis of 790 *Pf*AMA-1 sequences. Haplotype network analysis shows that haplotypes on Bioko Island are scattered among other haplotypes from different countries, which is consistent with a previous report [53]. Compared with the African continent, Bioko Island is an independent Island with a special geographical location. It is an offshore Island, with special species diversity and evolution, but very close to seriously affected areas of malaria in West Africa. As a result, the distribution of *P. falciparum* on Bioko Island may to have no differences with the continent of Africa, but the distribution of the different geographic strains on the island is unclear. Therefore, the genetic diversity of *Pf*AMA-1 DI regions on Bioko Island were analysed to carry out a detailed investigation on the distribution of different geographical strains to help understand the situation in this region and provide a reference for drug resistance investigations and medication guidance. Many single haplotypes appear in both clusters, but no pie completely covering all haplotypes was found in all geographic areas in this study. A recent report suggested that mutations in the *Pf*AMA-1 sequence are not necessarily strong predictors of antigenic differences or cross-inhibitory antibody activity levels, since not all polymorphic residues contribute equally to antibody generation and escape [47]. Due to the limited diversity of *Pf*AMA-1 antigens, vaccines targeting a small number of *Pf*AMA-1 alleles might be sufficient to cover naturally circulating populations of *P. falciparum* in different endemic areas [33]. These results also indicate that the global genetic diversity of *Pf*AMA-1 is relatively limited, even though substantial geographic differentiation can also be identified among populations. Nevertheless, global *Pf*AMA-1 is undergoing natural selection and high levels of meiotic recombination, which can produce new alleles in a gene population. Therefore, consideration should be given to the development of *Pf*AMA-1-based malaria vaccines using a polyallelic approach to maximize vaccine effectiveness.

To assess the association between host immune pressure and natural selection of *Pf*AMA-1, genetic polymorphisms in predicted B-cell epitopes and IUR regions were analysed. Based on a recent report, detailed information on potential RBC-binding regions, B-cell epitopes and IURs across the ectodomain of *Pf*AMA-1 were obtained [45]. Most amino acid changes found in DI of *Pf*AMA-1 on Bioko Island are predicted to be localized at predicted B-cell epitopes or IUR regions in DI. In this study, B-cell epitopes 3 and 4 show high levels of nucleotide diversity in *Pf*AMA-1 on Bioko Island. Tajima’s D values for these predicted B-cell epitopes also suggest that these epitopes are under natural selection. The π values of RBC-2 regions in different geographical areas are close to each other, which suggests that the nucleotide diversity of DIs is similar in these regions. In Bioko Island *Pf*AMA-1 isolates analysed in this study, the DI region shows clustering of amino acid polymorphisms in the C1L region. The C1L region is located near the hydrophobic pocket of DIs, which affects the binding of inhibitory monoclonal antibodies and thus leads to escape from antibody targeting [55, 56]. This shows that the important role of natural selection in generating *Pf*AMA-1 gene diversity is very obvious just as the effect of natural selection of *P. falciparum* on Bioko Island, and also supports the idea that natural selection can promote host immune escape in this region [57, 58]. Therefore, it may be necessary to consider the polymorphism in DI in order to obtain more efficient vaccine components.

## Conclusions

A major problem in the development of effective malaria vaccines is the genetic polymorphism observed in isolates worldwide. *Pf*AMA-1 is one of the most promising malaria vaccine candidates for the blood stage of *P. falciparum.* The overall pattern of nucleotide diversity and distribution of amino acid changes of *Pf*AMA-1 on Bioko Island is similar to those from other global isolates, although several novel amino acid changes are found on Bioko Island. Natural selection to balance across *Pf*AMA-1 and high levels of recombination events observed on Bioko Island and other global *Pf*AMA-1 genes suggest that natural selection and intragenic recombination might be the main drivers of genetic diversity in global *Pf*AMA-1. High levels of nucleotide diversity and natural selection suggest that strong natural selection might have an effect on immune stress-related epitopes of *Pf*AMA-1 hosts. The amino acid changes of DIs in *Pf*AMA-1 on Bioko Island are mainly in predicted B cell epitopes. Although a highly complex haplotype diversity has been found in global populations, genetic diversity of *Pf*AMA-1 between and among global parasite populations remains limited. Follow-up studies such as more samples and the expansion of *Pf*AMA-1 may yield more representative mutation trends. The results of this study ensure the continuous detection of *Pf*AMA-1 nucleotide and amino acid changes in global *Pf*AMA-1, so as to provide more useful information for the development of malaria vaccine.

## Supplementary information

**Additional file 1:** Amino acid sequence polymorphisms of domain I in *Pf*AMA-1 from Bioko Island isolates of *P. falciparum.*

**Additional file 2:** Recombination events in global *Pf*AMA-1 genes. Linkage disequilibrium (LD) plots showed a non-random association between nucleotide variants in DI of *Pf*AMA-1 at different polymorphic sites. R^2^ values were plotted against nucleotide distance using a two-tailed Fisher’s exact test for statistical significance.

## Data Availability

The datasets supporting the conclusions of this article are included with in the article.

## Abbreviations

*Pf*AMA-1: *Plasmodium falciparum* apical membrane antigen-1
DI: domain I
CSP: circumsporozoite protein
DBP: Duffy-binding protein
MSP-1: merozoite surface protein-1
TRAP: thrombospondin-related anonymous protein
BIMCP: The Bioko Island Malaria Control Project
S: numbers of segregating sites
H: haplotypes
Hd: haplotype diversity
π: nucleotide diversity
K: average number of pair-wise nucleotide differences within a population
dN: values of non-synonymous
dS: values of synonymous
R: recombination parameters
Rm: minimum number of recombination events
LD: linkage disequilibrium
Fst: fixation index
