# Supplementary material for: Natural selection and genetic diversity of domain I of Plasmodium falciparum apical membrane antigen-1 on Bioko Island
Source: Malar J. 2019 Sep 18;18:317. doi: 10.1186/s12936-019-2948-y (PMC6751645; doi:10.1186/s12936-019-2948-y)
Supplement: Supplementary file 1 — Additional file 1: Amino acid sequence polymorphisms of domain I in PfAMA-1 from Bioko Island isolates of P. falciparum. [file 12936_2019_2948_MOESM1_ESM.pdf]

	1	1	1	1	1	1	1	1	1	1	1	1	1	1	1	1	1	2	2	2	2	2	2	2	2	2	2	2	2	2	2	2	2	2	2	2	2	2	2	3
	4	5	5	5	6	6	7	7	7	8	8	8	8	9	9	9	9	0	0	0	0	0	2	2	3	4	4	4	4	6	6	6	7	8	8	8	8	8	9	0
	9	0	1	8	2	7	2	3	5	0	2	7	9	0	6	7	9	0	1	4	6	7	5	8	0	2	3	4	5	6	7	9	2	1	2	3	5	6	6	0
3D7(U65407)	C	P	V	I	N	T	G	N	Y	G	A	E	L	M	D	E	R	H	F	D	K	Y	I	N	K	D	K	D	K	D	E	K	S	D	I	S	Q	N	D	K
Haplotype_1													**H**										N					N			Q				**K**					
Haplotype_2					K		**E**		**D**			**N**	**P**		**N**	G		D	**L**	**N**	E		N		**E**										**K**					
Haplotype_3							**E**		**D**			**N**		I		H		D		**N**	E		N		**Q**	Y					Q				**K**					
Haplotype_4						K			**D**					I	**N**	G		D		**N**	E		N	K	**E**										**K**					
Haplotype_5					K		**E**		**D**							D		R			E		N			Y	**N**								**K**					
Haplotype_6									**D**						**N**	R		D		**N**	E		N	K	**E**	Y					Q				**K**					
Haplotype_7							**E**		**D**							Q															Q	I								
Haplotype_8					K		**E**		**D**			**N**	**P**		**N**	G		D	**L**	**N**	E		N			Y	**N**		**N**						**K**					
Haplotype_9					K		**E**		**D**			**N**	**P**		**N**	G		D	**L**	**N**	E		N		**E**			N			Q				**K**					
Haplotype_10								K	**D**			**N**		I	**N**	G	K	D			E	D	N	K	**E**	Y	**E**		**N**						**K**					
Haplotype_11							**E**		**D**			**N**		I	**N**	G	K	D			E	D	N	K	**E**	Y	**E**		**N**						**K**					
Haplotype_12						K			**D**							D		R		**N**	E		N			Y	**E**				Q				**K**	L	E			E
Haplotype_13								K	**D**			**N**		I	**N**	G	K	D			E	D	N	K	**E**	Y			**E**						**K**					
Haplotype_14							**E**		**D**							R		D		**N**	E	D	N					N			Q				**K**					E
Haplotype_15						K			**D**			**N**		I	**N**	G		D	**L**	**N**	E	D	N			Y	**N**								**K**					
Haplotype_16					K		**E**		**D**					I		Q			**L**		E		N			Y	**E**								**K**	L	E			E
Haplotype_17							**V**		**D**			**K**				D		L	**L**		E	D													**K**	L	E			
Haplotype_18					K		**E**		**D**			**N**	**P**			D		L	**L**		E	D	N												**K**					E
Haplotype_19					K		**E**		**D**			**N**	**P**			G		L	**L**		E	D	N												**K**					E
Haplotype_20							**E**		**D**			**K**		I		Q		D		**N**	E		N		**E**	Y					Q				**K**	L	E		H	
Haplotype_21					K		**E**		**D**			**N**	**P**			D		L	**L**		E	D	N												**K**					E
Haplotype_22						K			**D**					I	**N**	G		D		**N**	E		N	K	**E**	Y	**E**				Q				**K**					
Haplotype_23									**D**			**K**				D		L	**L**		E	D	N			Y	**N**				Q				**K**	L	E			E
Haplotype_24					K		**E**		**D**			**N**	**P**			D		L	**L**		E	D	N												**K**					
Haplotype_25						K			**D**					I		D		D		**N**	E		N			Y	**E**				Q				**K**	L	E			E
Haplotype_26													**H**													Y	**E**				Q				**K**	L				E
Haplotype_27							**V**		**D**			**K**				G		V	**L**		E	D													**K**	L	E			
Haplotype_28													**H**													Y	**E**				Q	I								
Haplotype_29									**D**					I		D		D		**N**	E		N		**Q**	Y					Q				**K**					
Haplotype_30							**E**		**D**							H		D		**N**	E		N		**Q**	Y	**E**				Q				**K**	L	E			E
Haplotype_31							**E**		**D**			**K**	**H**			D		L	**L**		E	D									Q				**K**			D		
Haplotype_32							**E**		**D**			**K**				H				**N**																				
Haplotype_33						K			**D**					I		D		D		**N**	E		N		**Q**	Y	**N**		**N**						**K**				H	
Haplotype_34							**E**		**D**			**K**				Q															Q	I								
Haplotype_35									**D**					I		D		D		**N**	E		N		**Q**	Y	**N**		**N**						**K**				H	
Haplotype_36												**K**				D		L	**L**		E	D													**K**				H	
Haplotype_37					K		**E**		**D**			**K**				D		R			E		N			Y	**N**								**K**					
Haplotype_38									**D**			**N**	**P**			Q															Q	I								
Haplotype_39																																			**K**					
Haplotype_40									**D**			**N**		I		G		D					N			Y					Q				**K**					
Haplotype_41					K		**E**		**D**	C		**N**	**P**		**N**	G		D	**L**	**N**	E		N			Y	**N**								**K**					
Haplotype_42									**D**			**N**		I	**N**	G	K	D		**N**	E		N		**E**	Y					Q				**K**					
Haplotype_43							**E**		**D**			**K**				Q		D		**N**	E		N		**Q**	Y	**E**				Q				**K**	L	E			E
Haplotype_44	*	T			K		**E**		**D**			**K**				D		L	**L**		E	D	N			Y	**N**				Q				**K**	L	E			E
Haplotype_45	K	T		S	K		**E**		**D**		V					D		R			E		N			Y	**N**								**K**					
Haplotype_46						K			**D**					I		D		D		**N**	E		N		**Q**	Y	**E**				Q				**K**	L			H	
Haplotype_47						K			**D**			**N**	**P**		**N**	C		D	**L**	**N**	E		N			Y	**E**				Q				**K**	L				E

Haplotype_48																																							
Haplotype_49							E		D		N		I		H		D	S	N	E		N		Q	Y					Q				K					
Haplotype_50					K		E		D				I		Q					E	D	N			Y	E								K	L	E			
Haplotype_51	*			S			E		D						D		G			E		N			Y	N								K					
Haplotype_52						K			D		N		I	N	C		D	L	N	E		N			Y	N		N						K					
Haplotype_53		T				K	E		D		N				H			L		E		N			Y	E				Q				K					
Haplotype_54	Y							K	D	C	N		I	N	G	K	D			E	D	N	K	E	Y									K					
Haplotype_55									D		K				D					E	D													K					
Haplotype_56						K			D	C			I	N	C		D		N	E		N	K	E										K					
Haplotype_57					K		E		D						D		L			E	D	N				E								K					E
Haplotype_58							E		D		K				Q																								
Haplotype_59						K			D				I	N	G		D		N	E		N	K	E										K	L				E
Haplotype_60									D		K		I		H		D		N	E		N			Y									K				H	
Haplotype_61							E		D		D				G		D		N	E		N			Y	E								K					
Haplotype_62																																		K					E
Haplotype_63							E		D		N				Q		D			E	D	N		E	Y									K					
Haplotype_64							E		D		K				Q										Y	E				Q	I								
Haplotype_65									D		K		I		H		D		N	E		N			Y					Q				K					
Haplotype_66							E		D		K		I		Q		D	L	N	E		N			Y	N		N		Q	I								
Haplotype_67							E		D		K		I		H			L		E		N			Y			N		Q	I								
Haplotype_68									D		N	P		N	G		D	L	N	E		N		E										K					E
Haplotype_69						K			D				I	K	G		D		N	E		N	K	E										K					
Haplotype_70									D		K				D		L	L		E	D	N			Y	N				Q		N		K	L	E			E
Haplotype_71							E		D		N				H				N	E		N		Q	Y					Q				K					
Haplotype_72					K		E		D		N	P		N	G		D	L	N	E		N		E										K					E
Haplotype_73								K	D		N		I	N	G		D	L	N	E	D	N			Y	N				Q				K	L	E			E
Haplotype_74					K		E		D		N	P		N	G		D	L	N	E		N		E						Q				K					
Haplotype_75								K	D		N		I	N	G	K				E	D	N	K	E	Y									K					
Haplotype_76												H													Y	E								K	L				E
Haplotype_77							E		D		K	H			Q										Y	E				Q				K	L	E			E
Haplotype_78						K			D		N		I		H		D		N	E		N			Y	E				Q				K	L			H	E
Haplotype_79							E		H						H		D		N	E		N		Q	Y	E				Q				K	L	E			E
Haplotype_80						K			D		N		I	N	G		D	L		E	D	N	K	E	Y	E		N					H	K					E
Haplotype_81						K			D				I		D		D		N	E		N		Q	Y	N		N	N					K				H	
Haplotype_82						K			D		N		I		Q					E	D	N					N							K			D		
Haplotype_83							E		D		K		I		Q		D		N	E		N		Q						Q				K				H	
Haplotype_84									D		K		I	N			D	L	N	E		N		E	Y					Q				K	L	E			
Haplotype_85							E		D						R		D		N	E	D									Q				K	L	E			
Haplotype_86																														Q				K					E
Haplotype_87							V		D		K				D		L	L		E	D	N								Q				K					E
Haplotype_88							E		D		K	H			D		L	L		E										Q				K			D		
Haplotype_89							E		D		K	H			D		L		N	E										Q				K					
Haplotype_90							E		D		K		I	N	G		D			E		N	K	E	Y	E							H	K	L	E			
Haplotype_91							E		D		K	H			D		L	L		E										Q				K					
Haplotype_92								K	D		N		I	N	G	K	D			E	D	N	K	E	Y									K				H	
Haplotype_93			I				E		D		K		I	N	D		D		N	E		N								Q				K					
Haplotype_94							E		D		K				D		D		N	E		N								Q				K					
Haplotype_95							E		D		K		I		D		V		N	E		N								Q				K					
Haplotype_96							E		D		K		I		D				N	E		N								Q				K					
Haplotype_97									D		K		I		D		L		N	E		N								Q				K					
Haplotype_98									D		K		I		D		D		N	E		N		Q						Q				K					

Haplotype_99	K		**E**		**D**			I		D		L	**L**		E	D	N			Y	**E**		Q		**K**	L			E
Haplotype_100					**D**	**K**		I		D		D		**N**	E		N		**Q**	Y			Q		**K**				
Haplotype_101			**E**		**D**										E								Q		**K**				
Haplotype_102		K			**D**			I		D		D		**N**	E		N		**Q**	Y			Q		**K**				
Haplotype_103					**D**			I		G		D		**N**	E		N		**Q**	Y			Q		**K**				
Haplotype_104			**E**		**D**					Q					E		N						Q		**K**				
Haplotype_105					**D**	**K**		I		D		D		**N**	E		N						Q		**K**				
Haplotype_106		K			**D**	**K**		I	**N**	G		L		**N**	E		N			Y			Q		**K**				
Haplotype_107		K	**E**		**D**			I	**N**	G		D		**N**	E		N		**Q**				Q		**K**				
Haplotype_108		K			**D**			I		G		D		**N**	E		N		**Q**	Y			Q		**K**				
Haplotype_109			**E**		**D**	**N**		I	**N**	G	K	D			E	D	N		**E**	Y					**K**				
Haplotype_110	K		**E**		**D**					D		R			E		N				**N**				**K**				
Haplotype_111				K	**D**	**N**			**N**	G	K	D			E		N		**E**						**K**				
Haplotype_112	K		**E**		**D**	**N**	**P**		**N**	G		D	**L**	**N**	E		N	K	**E**	Y					**K**				
Haplotype_113			**E**							Q													Q		**K**				
Haplotype_114		K			**D**	**K**		I		D		D		**N**	E		N		**Q**	Y			Q		**K**	L			
Haplotype_115	K		**E**		**D**	**K**				D		L	**L**		E	D									**K**	L	E		
Haplotype_116		K	**E**		**D**	**N**	**P**			Q															**K**	L	E		
Haplotype_117			**E**		**D**	**N**	**P**		**N**	G		D		**N**	E		N					N			**K**				
Haplotype_118		K			**D**	**K**		I	**N**	G		D		**N**	E		N		**Q**	Y			Q		**K**				
Haplotype_119					**D**	**K**		I	**N**	G		D		**N**	E		N			Y			Q		**K**				
Haplotype_120			**E**		**D**	**K**		I		G		L		**N**	E		N			Y			Q		**K**				
Haplotype_121	K		**E**		**D**	**N**	**P**			D		L	**L**		E	D	N						Q		**K**	L	E		
Haplotype_122				K	**D**	**N**		I		H		D		**N**	E		N		**E**	Y					**K**	L		H	E
Haplotype_123					**D**	**K**		I	**N**	G		D		**N**	E		N		**Q**	Y			Q		**K**				
Haplotype_124				K	**D**	**N**		I	**N**	G	K	D	**L**		E	D	N	K	**E**	Y					**K**				
Haplotype_125				K	**D**	**N**		I		H		D	**S**	**N**	E		N		**E**	Y			Q		**K**				E
Haplotype_126	K		**E**		**D**	**K**				D		D	**L**	**N**	E		N			Y	**E**								
Haplotype_127				K	**D**	**N**		I		H		D		**K**	E		N		**E**	Y			Q		**E**			H	
Haplotype_128			**E**		**D**	**K**				H		D		**N**			N				**N**								
Haplotype_129			**E**		**D**	**K**				H		D		**N**	E		N								**K**				
Haplotype_130			**E**		**D**					Q										Y			Q		**K**				
Haplotype_131			**E**		**D**					Q													Q	I	**K**				
