# Supplementary figures and images for: Natural selection and genetic diversity of domain I of Plasmodium falciparum apical membrane antigen-1 on Bioko Island
Source: Malar J. 2019 Sep 18;18:317. doi: 10.1186/s12936-019-2948-y (PMC6751645; doi:10.1186/s12936-019-2948-y)

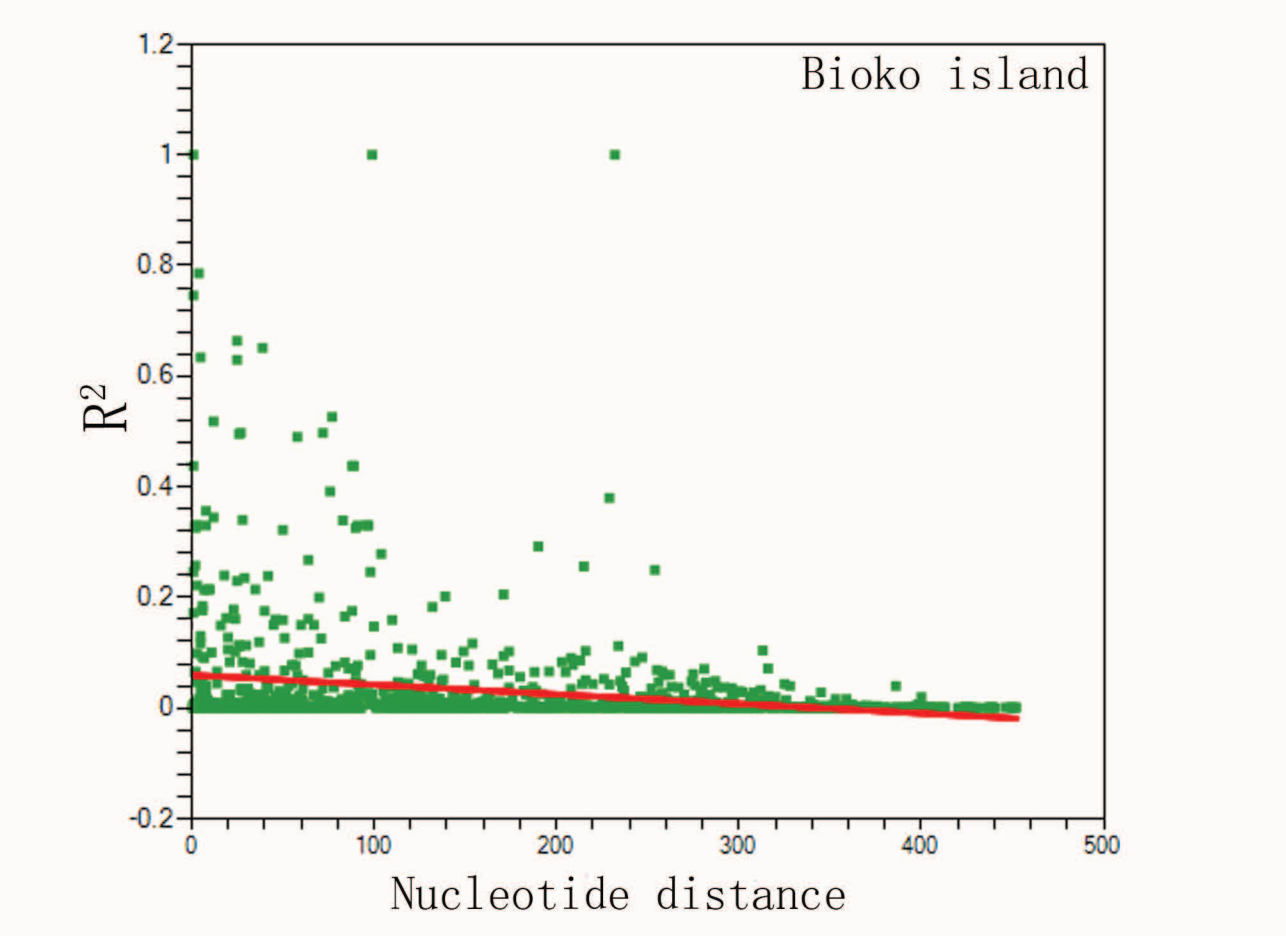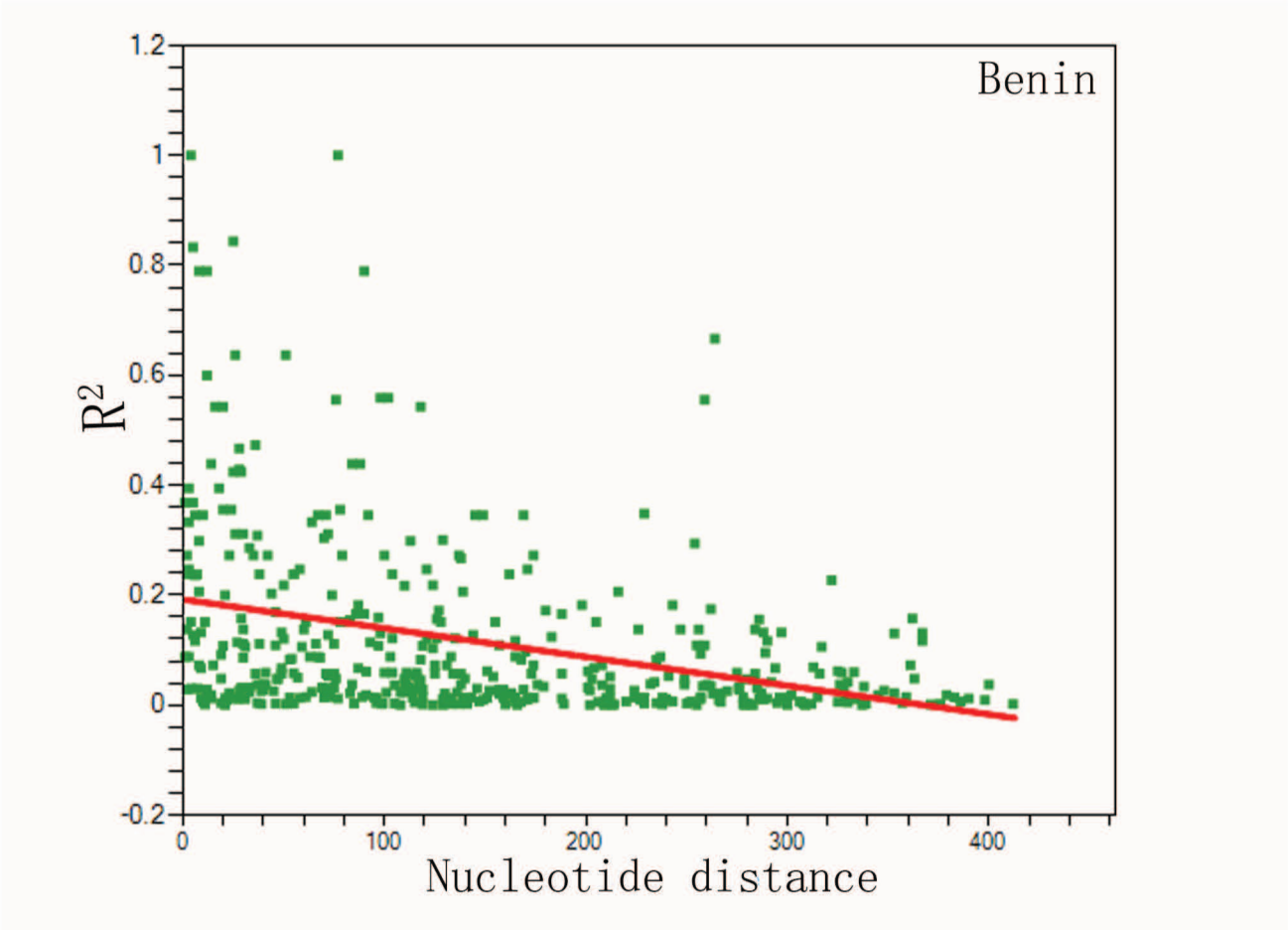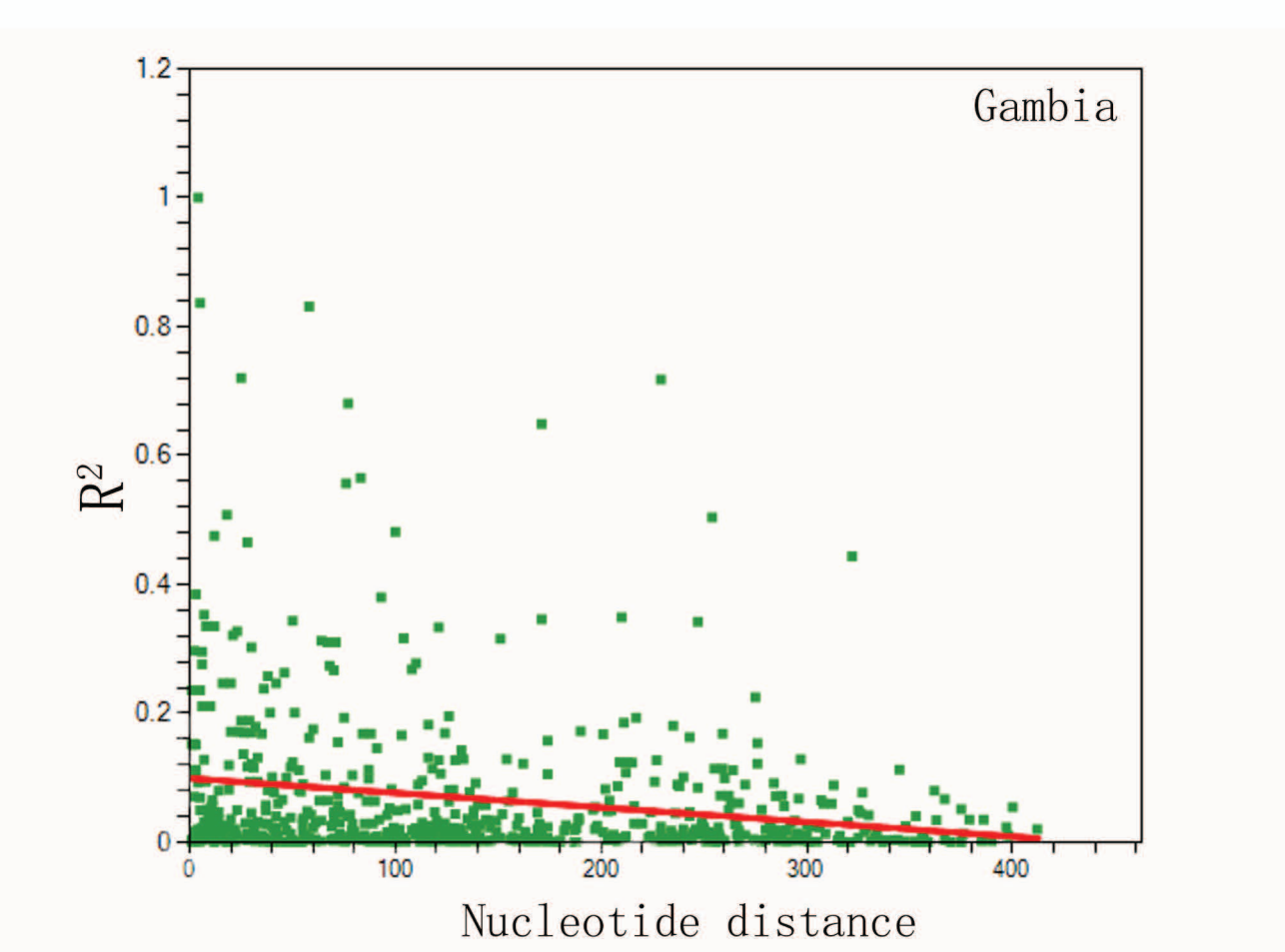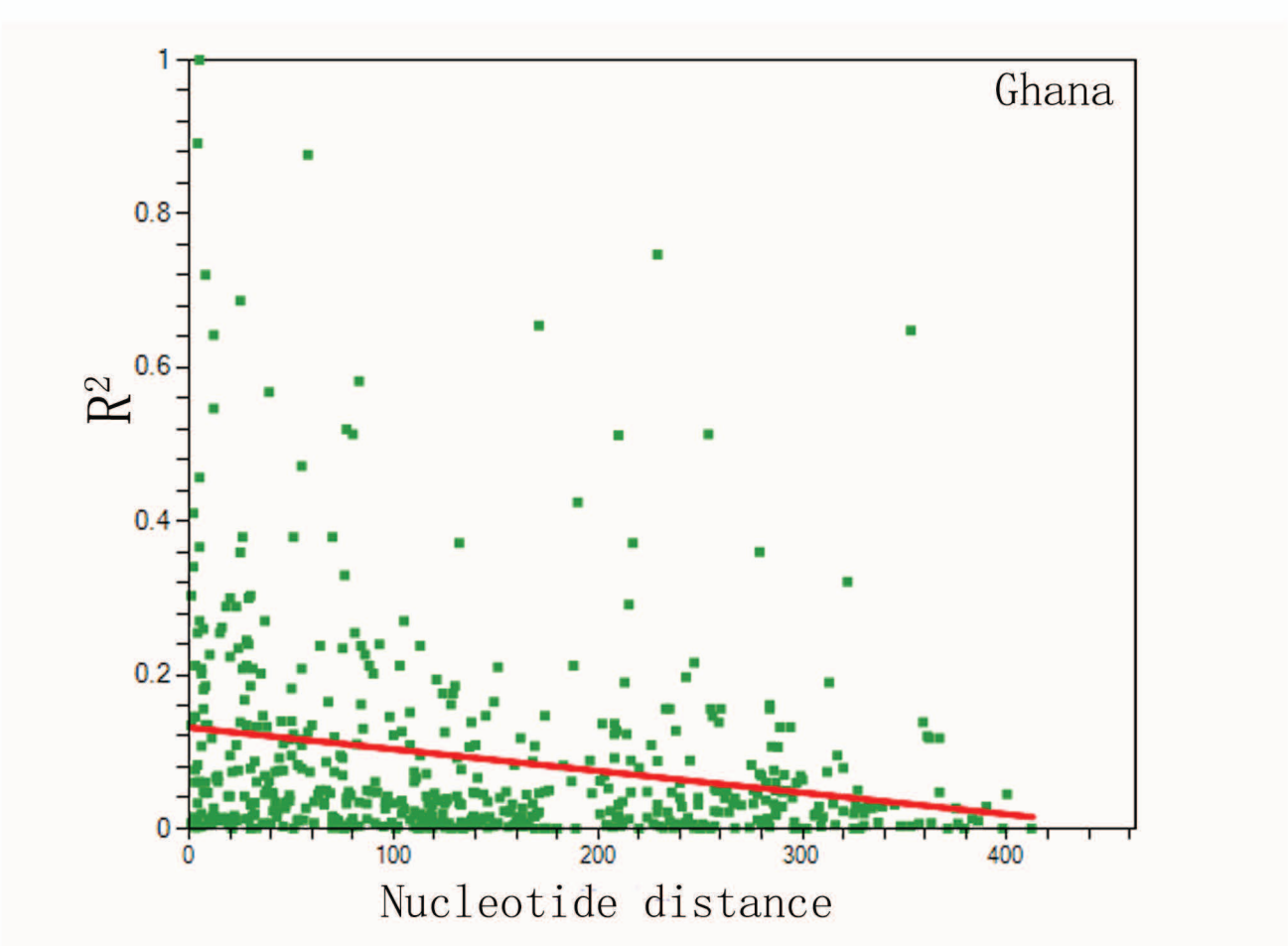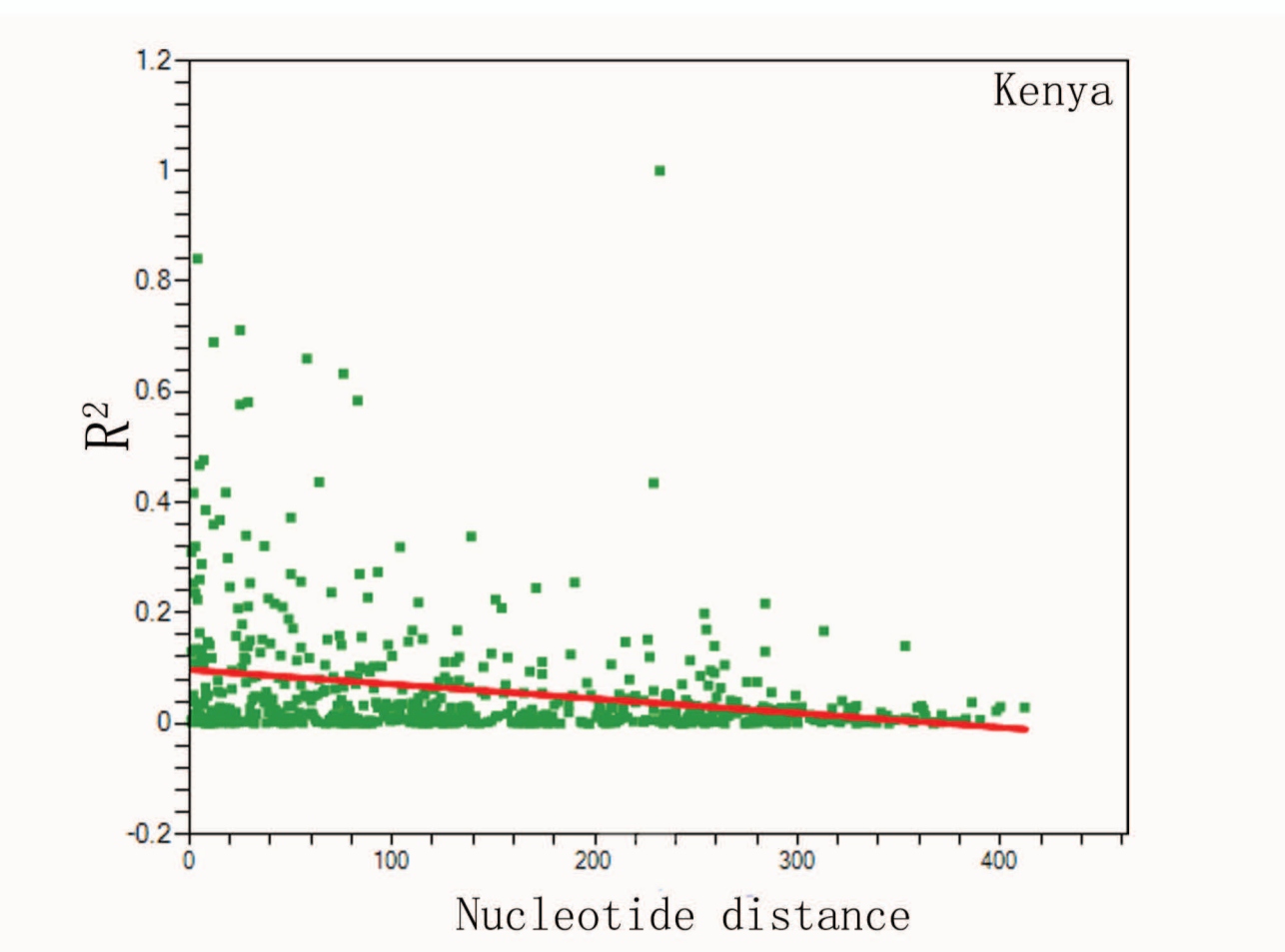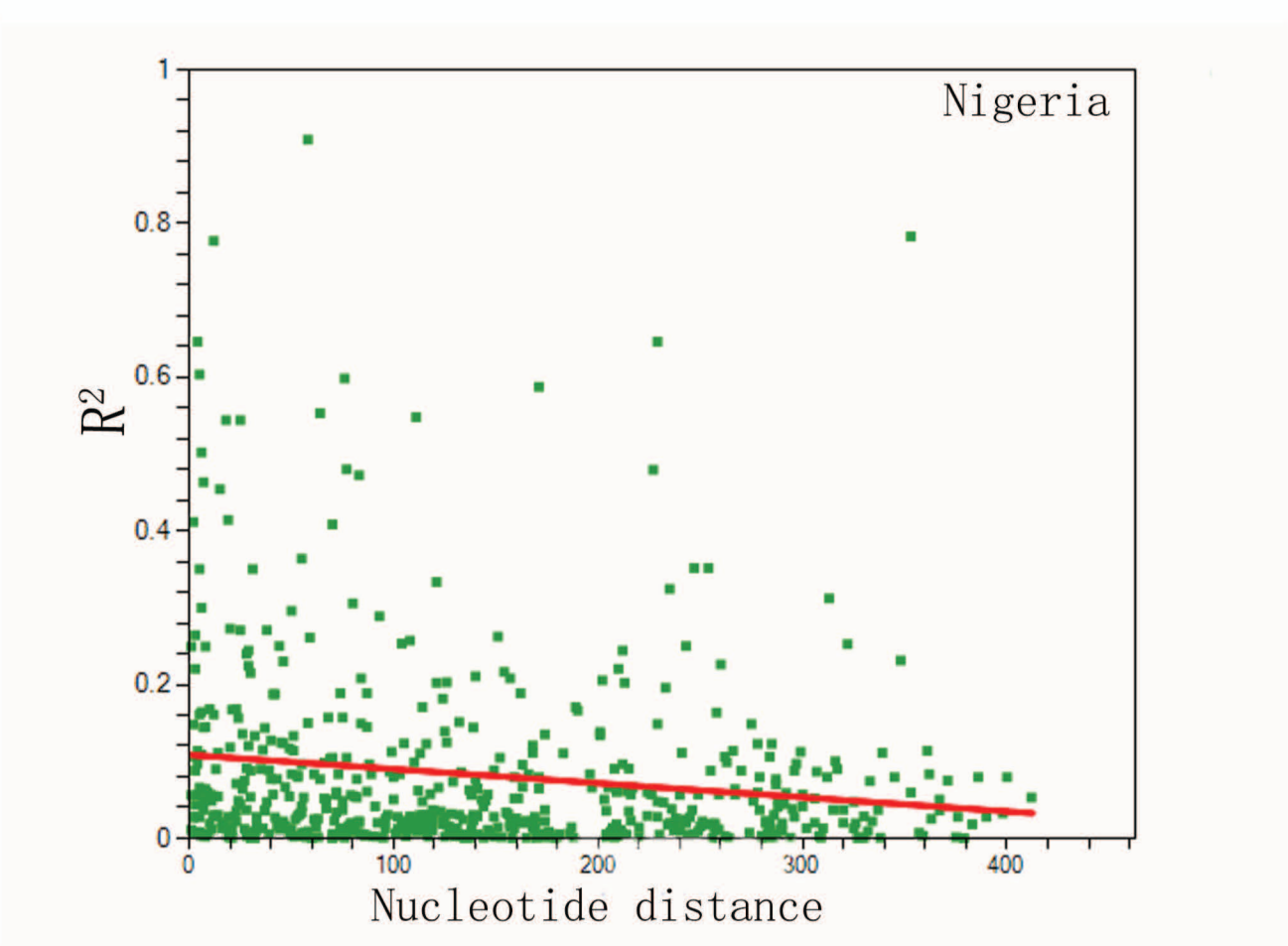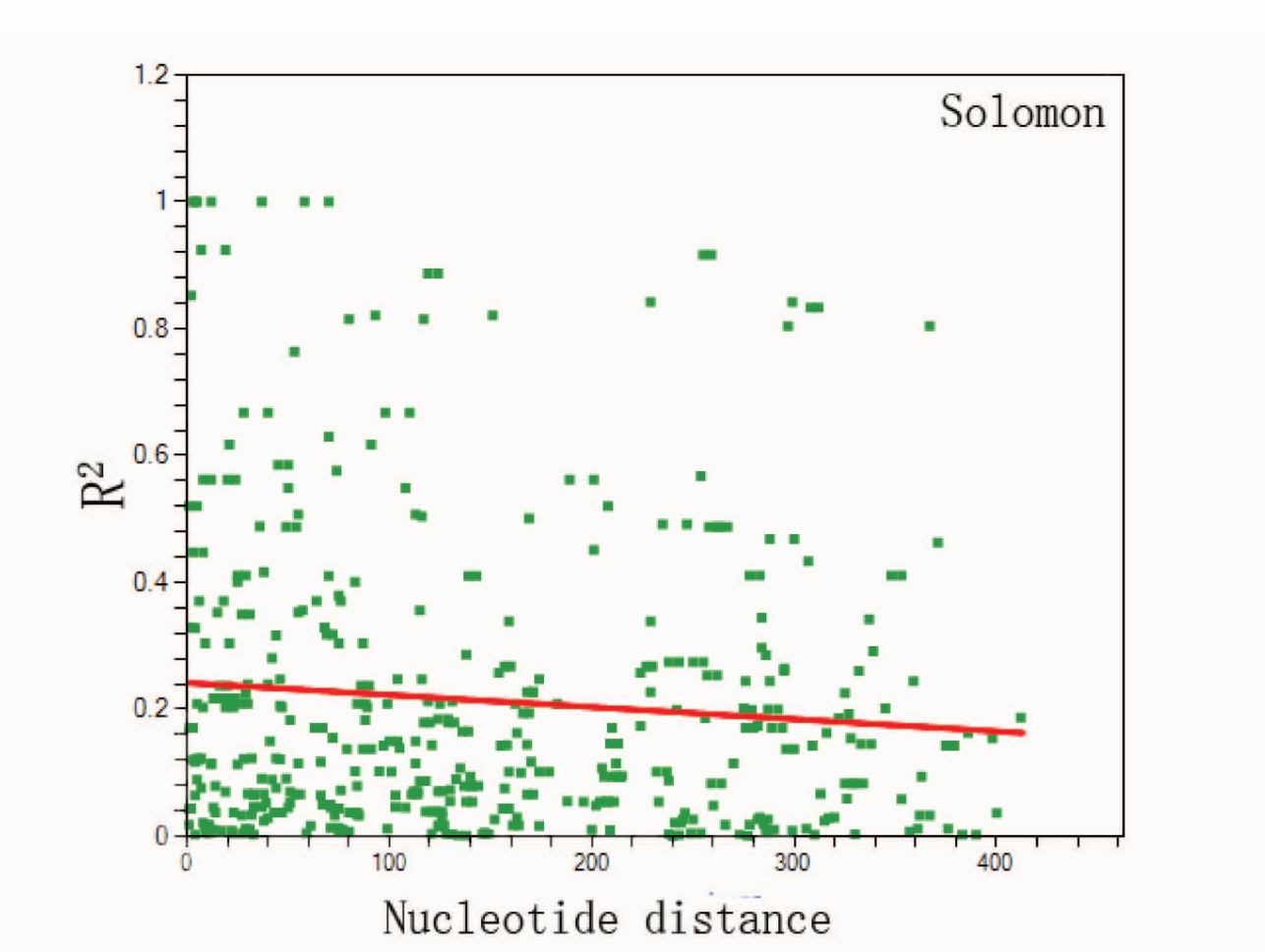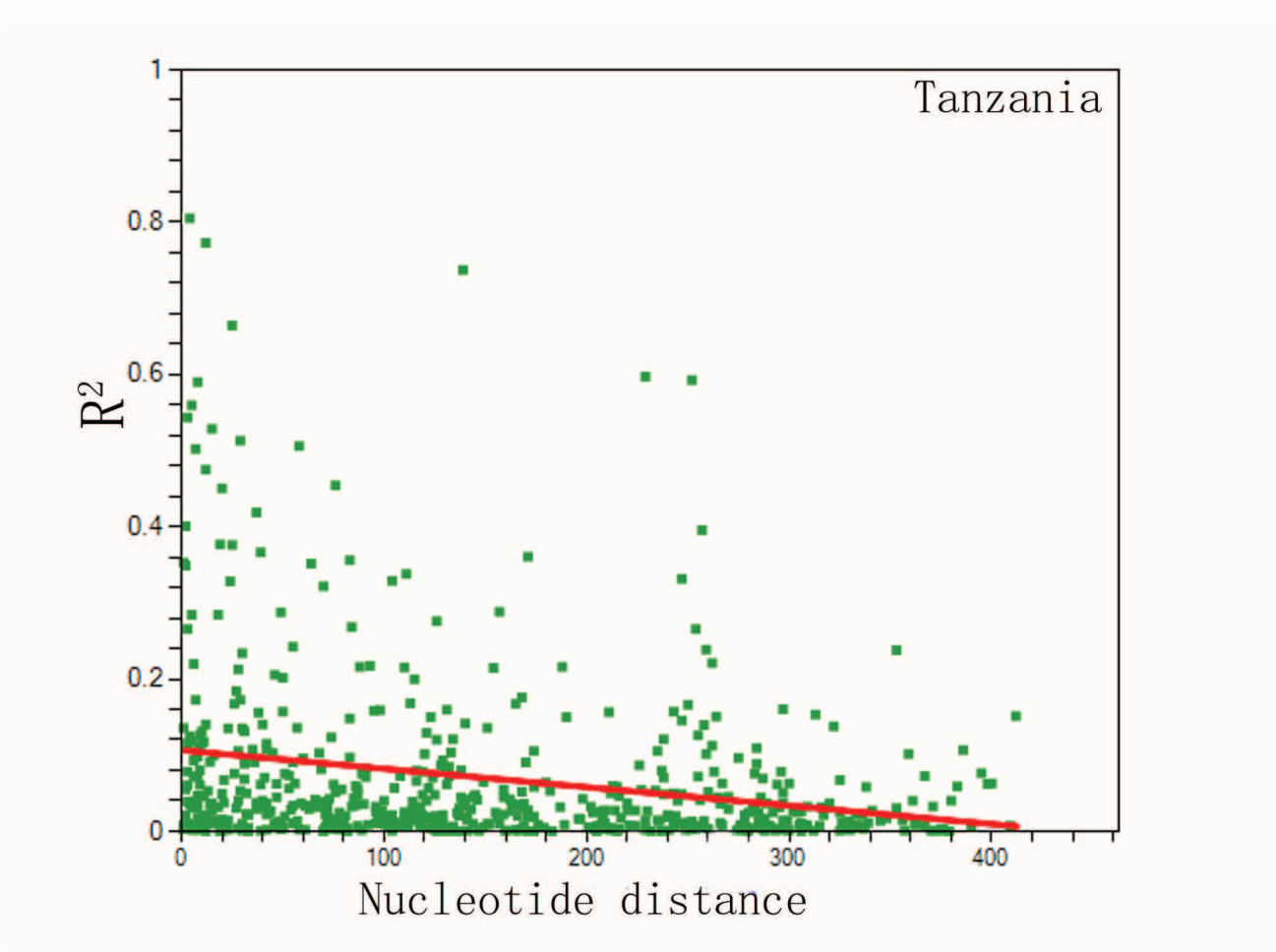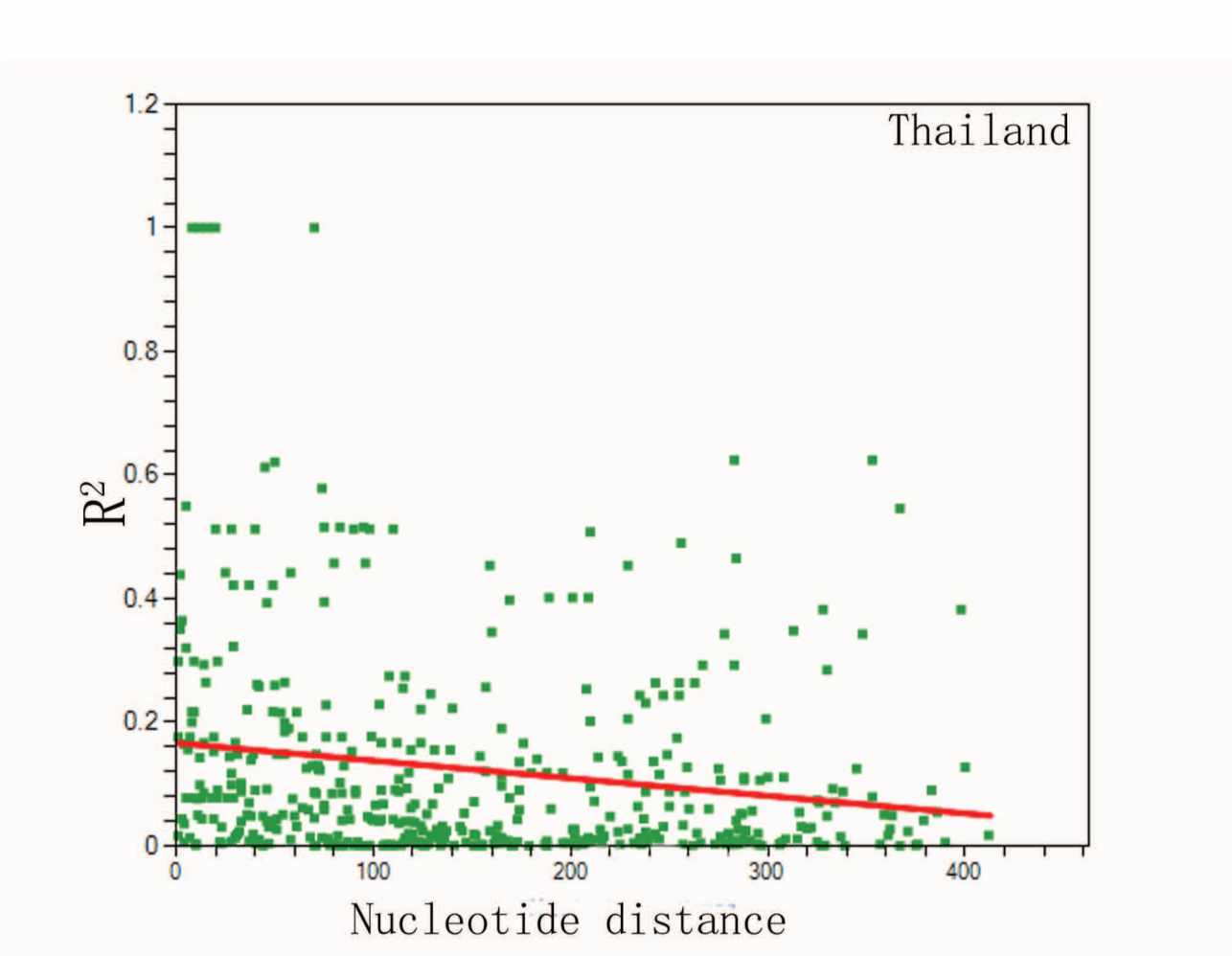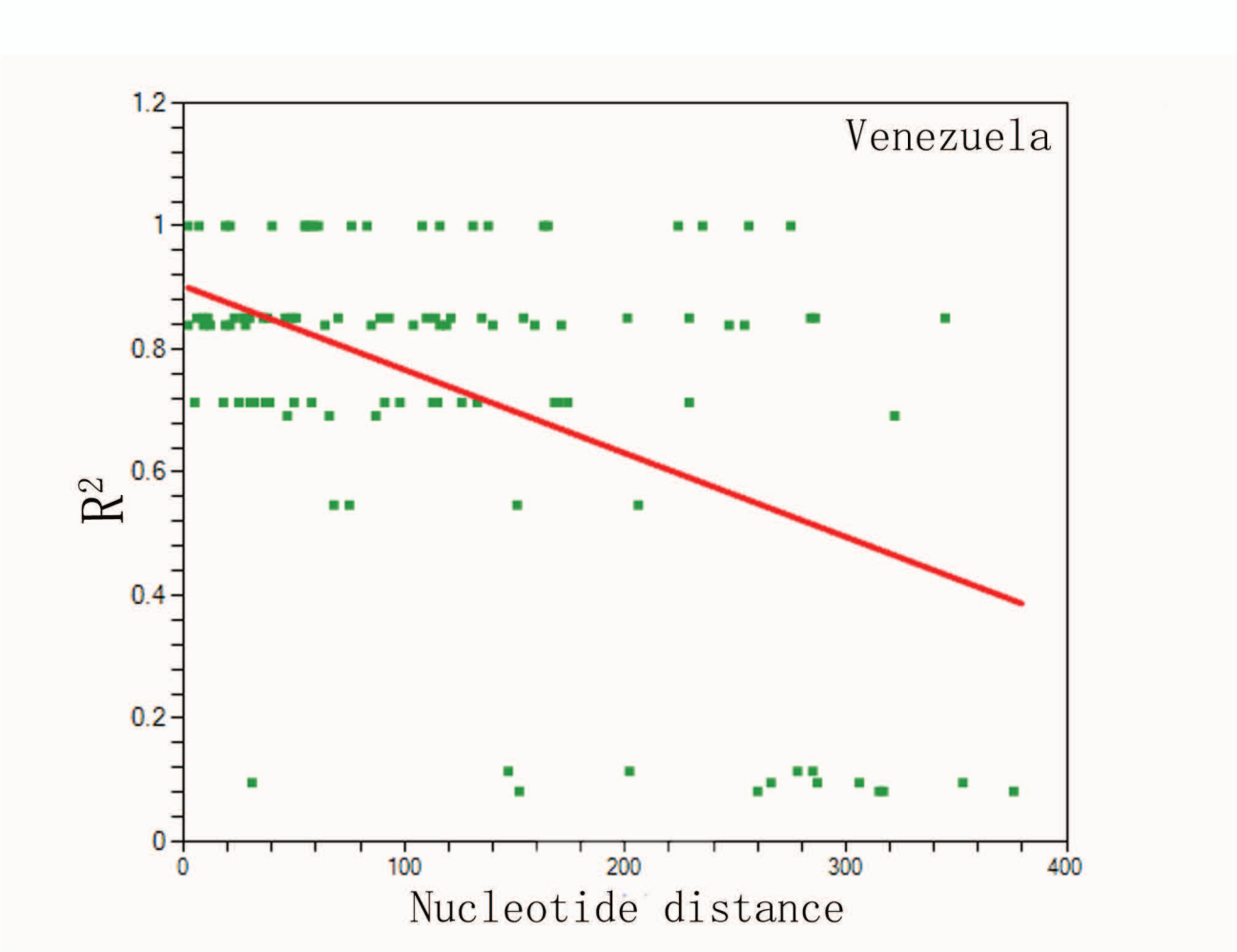

Supplement: Supplementary file 2 — Additional file 2: Recombination events in global PfAMA-1 genes. Linkage disequilibrium (LD) plots showed a non-random association between nucleotide variants in DI of PfAMA-1 at different polymorphic sites. R2 values were plotted against nucleotide distance using a two-tailed Fisher’s exact test for statistical significance. [file 12936_2019_2948_MOESM2_ESM.pdf]
